# CRY–BARs: Versatile light-gated molecular tools for the remodeling of membrane architectures

**DOI:** 10.1016/j.jbc.2022.102388

**Published:** 2022-08-18

**Authors:** Anna I. Wurz, Wyatt P. Bunner, Erzsebet M. Szatmari, Robert M. Hughes

**Affiliations:** 1 Department of Chemistry, East Carolina University, Greenville, North Carolina, USA; 2 Department of Physical Therapy, East Carolina University, Greenville, North Carolina, USA

**Keywords:** BAR domain, membrane dynamics, optogenetics, membrane remodeling, cryptochrome 2, spinogenesis, BAR, Bin, Amphiphysin, and Rvs, BSA, bovine serum albumin, DIV, day *in vitro*, DMEM, Dulbecco's modified Eagle's medium, DPBS, Dulbecco’s PBS, FBS, fetal bovine serum, HEK293T, human embryonic kidney 293T cell line, I-BAR, inverse BAR, MTSS1, Missing in Metastasis 1, PIP2, phosphatidylinositol 4,5-bisphosphate, TBS, Tris-buffered saline, TBST, TBS with Tween-20

## Abstract

BAR (Bin, Amphiphysin, and Rvs) protein domains are responsible for the generation of membrane curvature and represent a critical mechanical component of cellular functions. Thus, BAR domains have great potential as components of membrane-remodeling tools for cell biologists. In this work, we describe the design and implementation of a family of versatile light-gated I-BAR (inverse BAR) domain containing tools derived from the fusion of the *Arabidopsis thaliana* cryptochrome 2 photoreceptor and I-BAR protein domains (“CRY–BARs”) with applications in the remodeling of membrane architectures and the control of cellular dynamics. By taking advantage of the intrinsic membrane-binding propensity of the I-BAR domain, CRY–BARs can be used for spatial and temporal control of cellular processes that require induction of membrane protrusions. Using cell lines and primary neuron cultures, we demonstrate here that the CRY–BAR optogenetic tool evokes membrane dynamic changes associated with cellular activity. Moreover, we provide evidence that ezrin, an actin and phosphatidylinositol 4,5-bisphosphate–binding protein, acts as a relay between the plasma membrane and the actin cytoskeleton and therefore is an important mediator of switch function. Overall, we propose that CRY–BARs hold promise as a useful addition to the optogenetic toolkit to study membrane remodeling in live cells.

Membrane-bound architectures, including filopodia, lamellipodia, and dendritic spines in neurons, are critical for a cell’s ability to transmit and respond to extracellular cues. As such, chemists and biologists have developed numerous optogenetic and chemo-optogenetic tools to control cellular architectures *via* manipulation of plasma membrane dynamics (1, 2, 3). However, these tools have to date incorporated a relatively small fraction of the numerous proteins involved in the building and dismantling of these architectural features. In particular, nonenzymatic proteins have been overlooked: while numerous optogenetic strategies exist for enzyme-mediated control of membrane dynamics (4, 5, 6, 7, 8, 9), far fewer have incorporated nonenzymatic proteins as the basis of optogenetic switch action (10). As mechanical control of membrane architecture by nonenzymatic proteins is a critical component of cellular signaling (11), addressing this oversight will be a bridge to a more comprehensive understanding of cellular dynamics and function.

In recent years, the critical role of proteins involved in membrane curvature inducing and sensing has come to light (12). In particular, BAR (Bin, Amphiphysin, and Rvs) domain–containing proteins possess diverse activities at the plasma membrane (13, 14, 15, 16, 17, 18, 19); as such, a heightened understanding of these roles has invited recent investigations of their suitability for control with optogenetic tools. In a recent work, Jones *et al.* (20) used a two-component approach (iLID/SspB) to design a light-activated switch enabling recruitment of the extended Fes–CIP4 homology BAR domain from FBP17 to the plasma membrane and a hybrid two-component approach (iLID/SspB/pdDronpa1) enabling recruitment of the inverse BAR (I-BAR) domain from IRSp53 to the plasma membrane. These switches, designed to have minimal interaction with the cell membrane in the absence of light, promoted either positive (Fes–CIP4 homology BAR; FBP17) or negative (I-BAR; IRSp53) membrane curvature in response to light activation.

The MTSS1 (Missing in Metastasis 1) protein is considered the prototype of I-BAR proteins (21), but, to the best of our knowledge, has not previously been applied in an optogenetic context. In this work, we investigated the potential of the MTSS1 I-BAR domain to serve as an actuator of membrane architecture and plasma membrane dynamics. We demonstrate that the I-BAR domain from MTSS1, in conjunction with the Cry2 photoreceptor protein (22, 23, 24, 25), forms the basis of a versatile optogenetic approach (“CRY–BAR”) for controlling membrane dynamics and cellular architecture. In contrast to previous approaches, we have not sought to minimize the interaction between our constructs and the plasma membrane. Instead, CRY–BAR combines the intrinsic membrane-binding affinity of the I-BAR domain (26) with the homo-oligomerizing capability of Cry2 (22), resulting in a membrane-localized switch that induces membrane remodeling in response to blue light. We also provide insight into the mode of action of CRY–BAR by showing that ezrin, a membrane- and cytoskeletal-relay protein, is linked to the ability of CRY–BAR to induce light-activated membrane remodeling and restriction of cellular dynamics by acting as a relay between CRY–BAR activation and the actin cytoskeleton.

## Results and discussion

**Figure 1:**
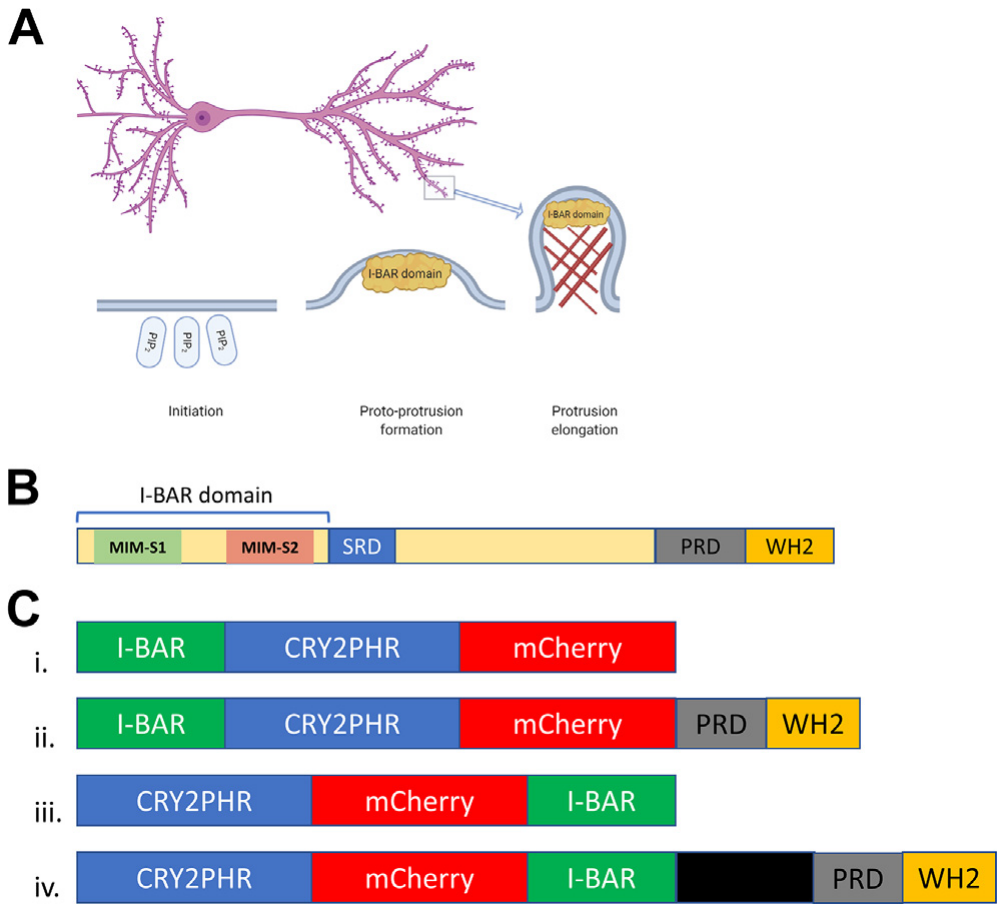
**I-BAR-mediated initiation ofdendritic spine generation in neurons.***A*, phosphoinositide (PIP2) signaling recruits I-BAR domain proteins to the plasma membrane, inducing a proto-protrusion. Subsequent recruitment of actin (*brown crosshatches*) and actin-binding and remodeling proteins promotes protrusion elongation. Mature dendritic spines (*purple nodules* on dendrite) are mushroom-shaped bulbous protrusions and major sites of excitatory synaptic transmission in the mammalian brain. *B*, diagram of MTSS1, an I-BAR domain–containing protein. The N-terminal I-BAR domain (250 amino acids) is comprised of three alpha helices. MIM-S1 and MIM-S2 represent the I-BAR domain dimerization interface as determined from X-ray crystallography (40). *C*, schematic of CRY–BAR optogenetic switches: (i) The N-terminal I-BAR domain (250 amino acids) is fused to photoreceptor protein CRY2, which is fused to the red fluorescent mCherry protein for visualization purposes. (ii) At the C terminus, proline-rich and WH2 domains can be included for actin recruitment. (iii) The N-terminal I-BAR domain is fused to the C terminus of mCherry. (iv) The intact MTSS1 protein (*green*, I-BAR; *black*, internal domains as shown in panel *B*; and *yellow*, WH2) is fused to the C terminus of mCherry. Graphic (*A*) generated with BioRender (https://biorender.com/). BAR, Bin, Amphiphysin, and Rvs; I-BAR, inverse BAR; MTSS1, Missing in Metastasis 1; PIP2, phosphatidylinositol 4,5-bisphosphate; PRD, proline-rich domain; SRD, serine-rich domain; WH2, Wiskott–Aldrich syndrome homology region.

Increased MTSS1 activity has been associated with exercise-induced enhancement of synaptic function (27). This prosynaptic plasticity effect of MTSS1 is attributed to the presence of an I-BAR domain within its structure. Moreover, I-BAR domains have also been shown to promote formation and stabilization of dendritic spines (Fig. 1*A*), leading to improved synaptic function and resilience against neurodegeneration (16). To gain insights into the molecular mechanisms that control membrane expansion associated with cell morphology changes, we fused the I-BAR domain of MTSS1 with a blue light–sensing domain (Cry2PHR; Fig. 1, *B* and *C*), creating a light-activatable I-BAR protein (CRY–BAR). For this investigation, we engineered four permutations of the CRY–BAR switch: I-BAR–Cry2–mCh, I-BAR–Cry2–mCh–WH2, Cry2–mCh–I-BAR, and Cry2–mCh–MTSS1 (Fig. 1*C*), where I-BAR = residues 1 to 250 of MTSS1, WH2 = residues 601 to 759 of MTSS1, and MTSS1 = the intact 759 residue protein. Each construct was confirmed by Sanger and NextGen sequencing and expressed predominantly at the expected molecular weight (Fig. S1).

**Figure 2:**
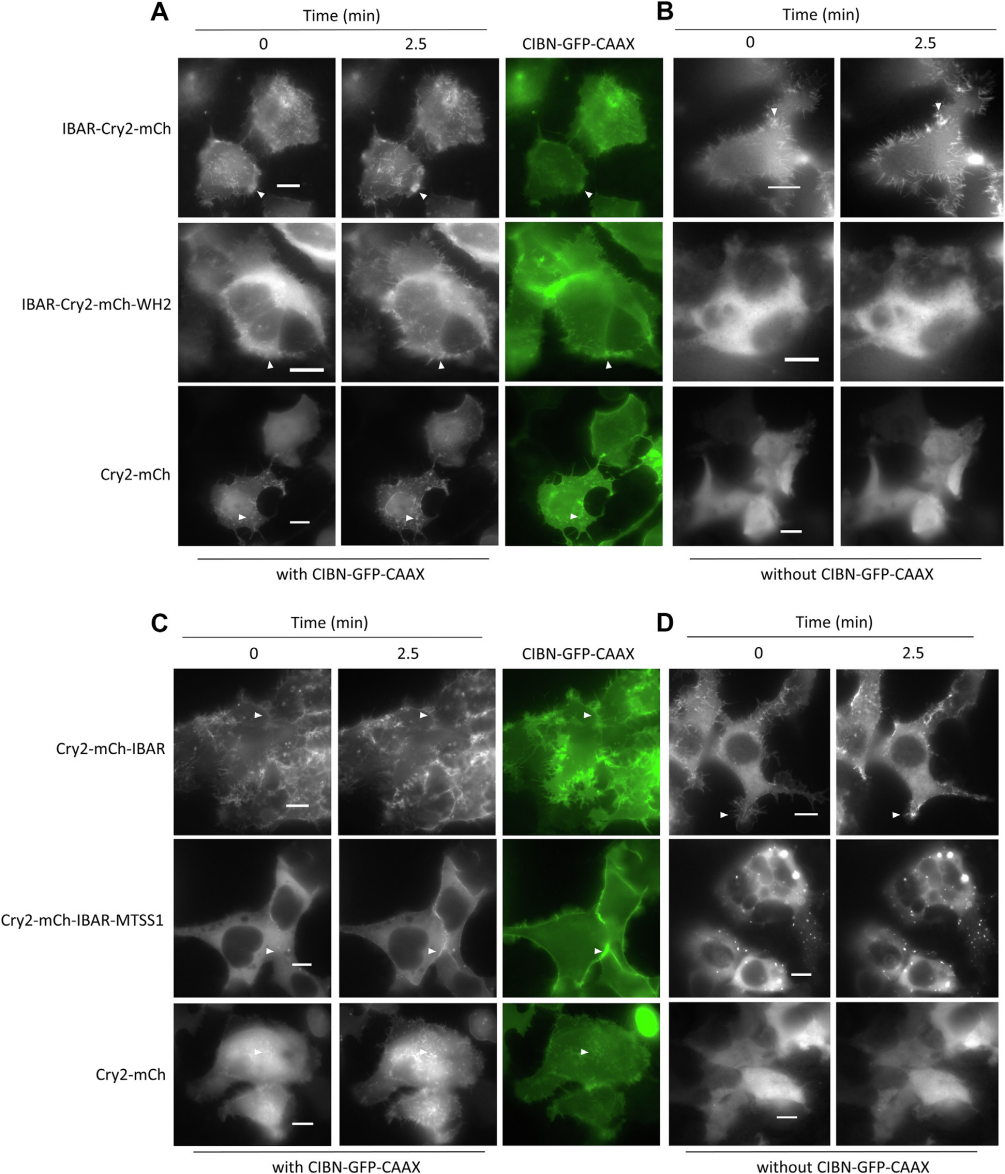
**Light-activated responses of CRY–BAR constructs.***A*, HEK293T cells were cotransfected with CIBN–GFP–CAAX and CRY–BAR fusions I-BAR–Cry2–mCh or I-BAR–Cry2–mCh–WH2 or Cry2–mCh and imaged on a widefield fluorescent microscope. I-BAR labeled as IBAR in figure. *B*, HEK293T cells were transfected with CRY–BAR fusions I-BAR–Cry2–mCh and I-BAR–Cry2–mCh–WH2 or Cry2–mCh and imaged on a widefield fluorescent microscope. *C*, HEK293T cells were cotransfected with CIBN–GFP–CAAX and CRY–BAR fusions Cry2–mCh–I-BAR and Cry2–mCh–MTSS1 or Cry2–mCh and imaged on a widefield fluorescent microscope. *D*, HEK293T cells were transfected with CRY–BAR fusions Cry2–mCh–I-BAR and Cry2–mCh–MTSS1 or Cry2–mCh and imaged on a widefield fluorescent microscope. Images acquired every 30 s (50 ms exposure of 480 nm light and 200 ms exposure of 553 nm light every 30 s). *White arrows* indicate example areas of light-activated recruitment. The scale bars represent 10 microns. BAR, Bin, Amphiphysin, and Rvs; HEK293T, human embryonic kidney 293T cell line; I-BAR, inverse BAR; MTSS1, Missing in Metastasis 1.

**Figure 3:**
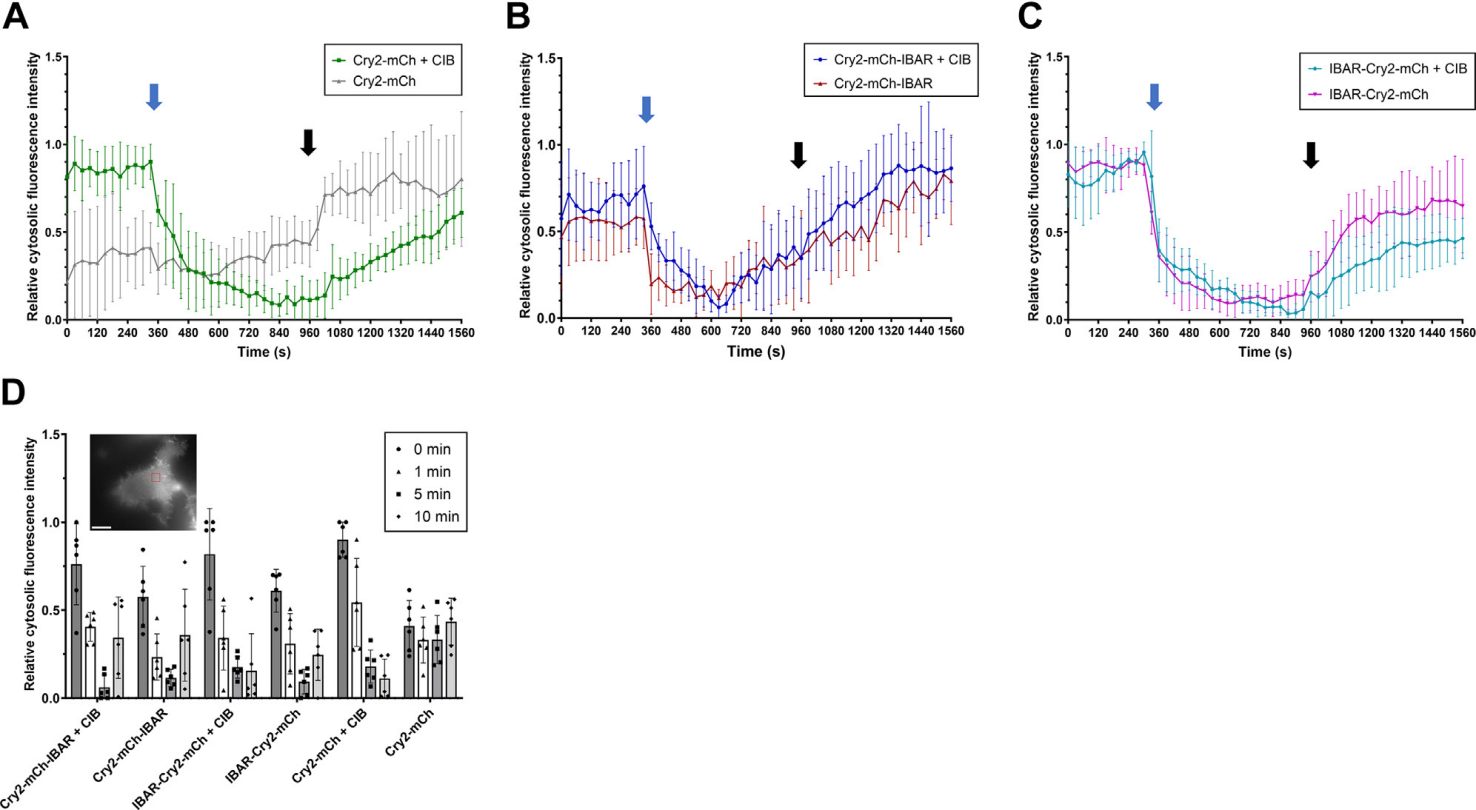
**Analysis of light-activated responses of CRY–BAR constructs.***A*, Cry2–mCh construct shows large decrease in cytosolic fluorescence intensity in the presence of plasma membrane–anchored CIBN–GFP–CAAX (CIB) but not in its absence. *B*, Cry2–mCh–I-BAR construct shows a decrease in cytosolic fluorescence intensity in both the presence and absence of plasma membrane–anchored CIB, indicative of significant homo-oligomerization at the plasma membrane. *C*, I-BAR–Cry2–mCh construct shows a decrease in cytosolic fluorescence intensity in both the presence and absence of plasma membrane–anchored CIBN, indicative of significant homo-oligomerization at the plasma membrane. *Blue arrows* indicate beginning of blue light illumination (50 ms exposure of 480 nm light every 30 s); *black arrows* indicate end of blue light illumination. Graphs show changes in normalized cytosolic mCherry fluorescence in HEK293T cells. Cells were initially imaged on mCherry channel (0–300 s), illuminated with 470 nm light for 10 min (330–930 s), then imaged on mCherry channel again for 10 min (960–1560 s). *D*, quantitation of the fluorescence of ROIs (n = 6 cells) at the time points: immediately before light exposure (0 min), then 1 min, 5 min, and 10 min of light exposure. *Inset* cell image: representative ROI inside the cytosol of HEK293T cell transfected with I-BAR–Cry2–mCh; this image is duplicated from Figure 2*A* to demonstrate measurement methodology. The scale bar represents 10 microns; ROI dimensions: 4.02 × 4.02 microns. I-BAR labeled as IBAR in figure.. BAR, Bin, Amphiphysin, and Rvs; HEK293T, human embryonic kidney 293T cell line; I-BAR, inverse BAR; ROI, region of interest.

CRY–BAR constructs were initially probed for their response to blue light in the presence of CIBN–CAAX, a membrane-localized binding partner to Cry2, in human embryonic kidney 293T (HEK293T) cells. As anticipated, light activation of the Cry2–mCh control resulted in rapid recruitment to the plasma membrane (Figs. 2*A* and 3*A*). Light-activated recruitment to CIBN–CAAX was also observed for all the I-BAR-containing constructs (Figs. 2 and 3, *B* and *C*). Interestingly, the I-BAR domain–containing constructs I-BAR–Cry2–mCh and Cry2–mCh–I-BAR, while exhibiting light-activated recruitment to the plasma membrane (Fig. 2, *A* and *C*), had significant prelocalization to the plasma membrane in the dark. We attributed this to the phosphatidylinositol 4,5-bisphosphate (PIP2)–binding propensity of the I-BAR domain (readily observed in the preillumination images in Fig. 2, *A* and *C*). Because of the significant prelocalization of these constructs to the plasma membrane, we then investigated whether they might exhibit light-activated recruitment, *via* Cry2 homo-oligomerization, in the absence of CIBN–CAAX. These experiments revealed significant light-activated clustering/membrane recruitment (Fig. 2, *B* and *D*) in the absence of CIBN–CAAX. By contrast, Cry2–mCh–MTSS1, which also accumulated in nonlight responsive clusters, did not exhibit light-activated membrane recruitment in the absence of CIBN–CAAX (50 of 50 cells pooled from six replicates; Fig. 2*D*). As a result, this construct was not pursued further.

**Figure 4:**
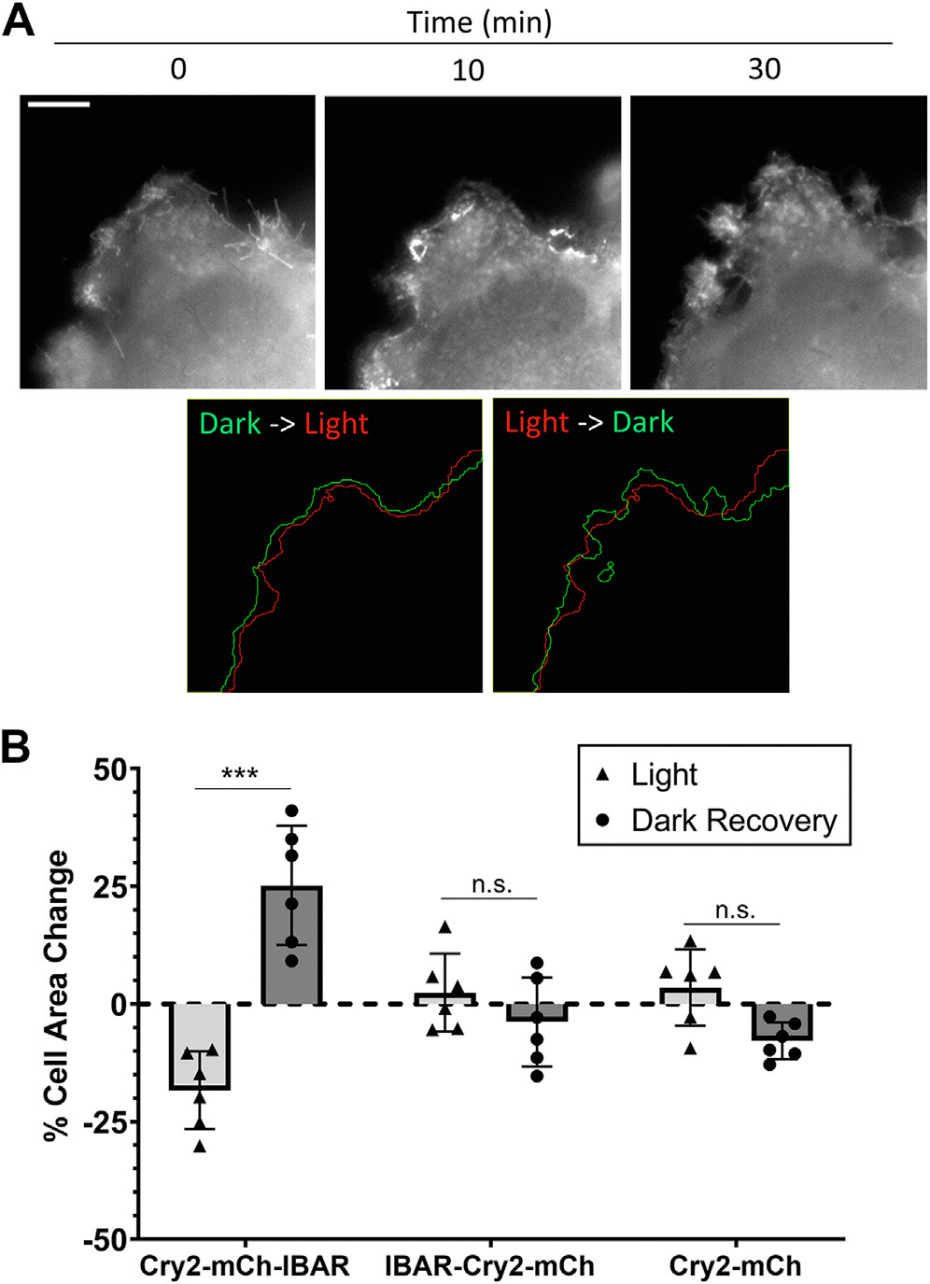
**Reversible light-activated membrane remodeling with CRY–BAR.***A*, HEK293T cells transfected with Cry2–mCh–I-BAR were subjected to blue light illumination for 10 min (activation), followed by 30 min without blue light (dark recovery). Membrane retraction and reshaping is observed during blue light illumination, followed by recovery in the absence of blue light. The scale bar represents 10 microns. *B*, quantification of changes in percent cell area during alternating cycles of light activation (10 min) and dark recovery (30 min) from n = 6 cells per construct pooled from three replicate measurements. Statistical analysis conducted with one-way ANOVA (∗∗∗*p* < 0.001; n.s., not significant [*p* > 0.05]). I-BAR labeled as IBAR in figure. BAR, Bin, Amphiphysin, and Rvs; HEK293T, human embryonic kidney 293T cell line; I-BAR, inverse BAR.

**Figure 5:**
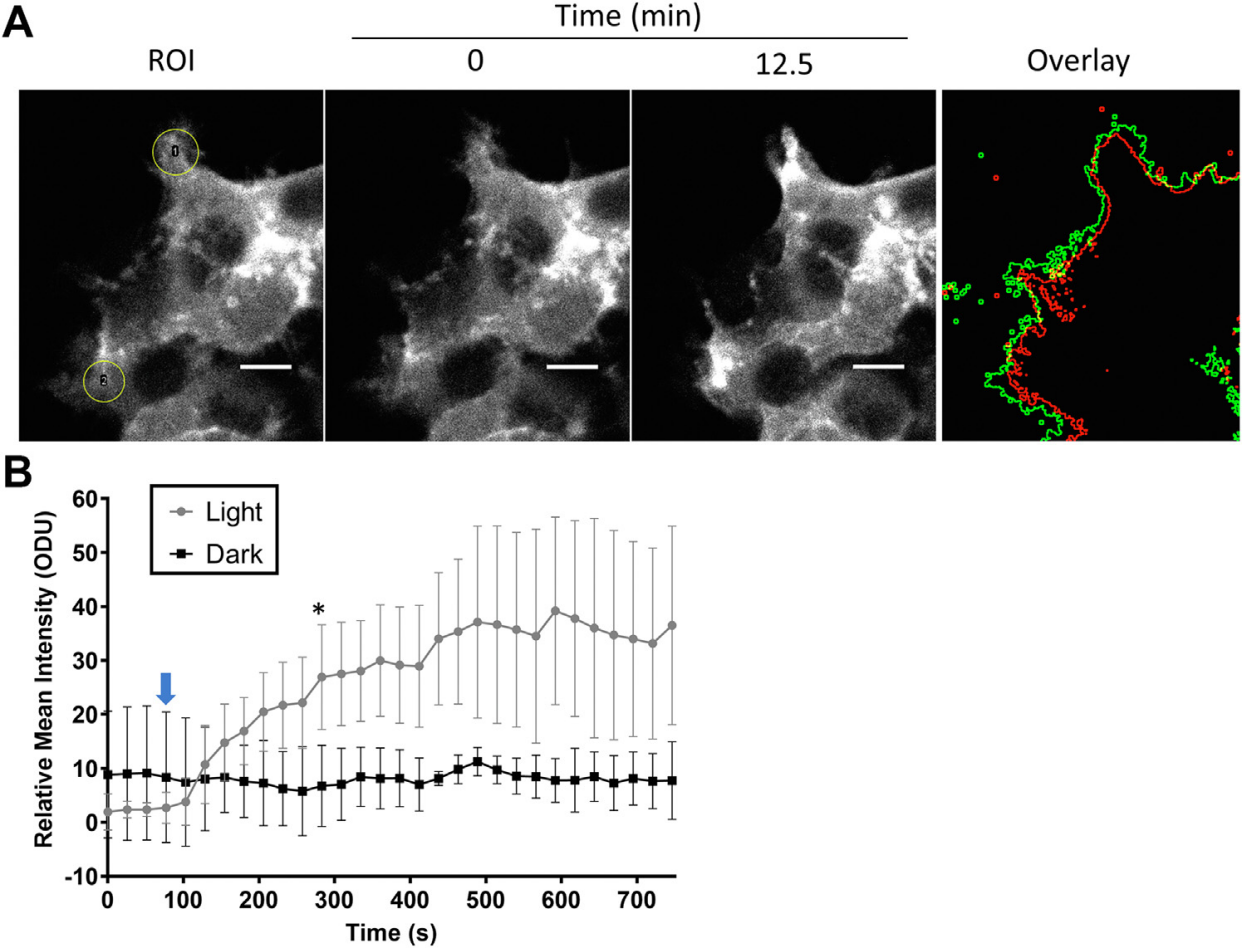
**Localized light activation of membrane remodeling with CRY–BAR.***A*, HEK293T cells transfected with Cry2–mCh–I-BAR were subjected to restricted blue light illumination (*yellow circles*) using a confocal microscope. Clustering of Cry–BAR is apparent in the areas illuminated 25 frames post–blue light stimulation. An overlay of the cell outline is shown before (*green*) and after (*red*) blue light illumination. The scale bar represents 10 microns. *B*, quantification of fluorescence intensity changes in illuminated *versus* nonilluminated cell areas for n = 3 circular ROIs (area = 61.4 microns^2^) per condition. *Blue arrow* indicates time of applied restricted blue light illumination (488 nm laser at 5% power). Error bars represent standard deviations. Differences are statistically significant (one-way ANOVA, *p* < 0.05) beginning at the 283 s time point (∗) and beyond. BAR, Bin, Amphiphysin, and Rvs; HEK293T, human embryonic kidney 293T cell line; I-BAR, inverse BAR; ROI, region of interest.

**Figure 6:**
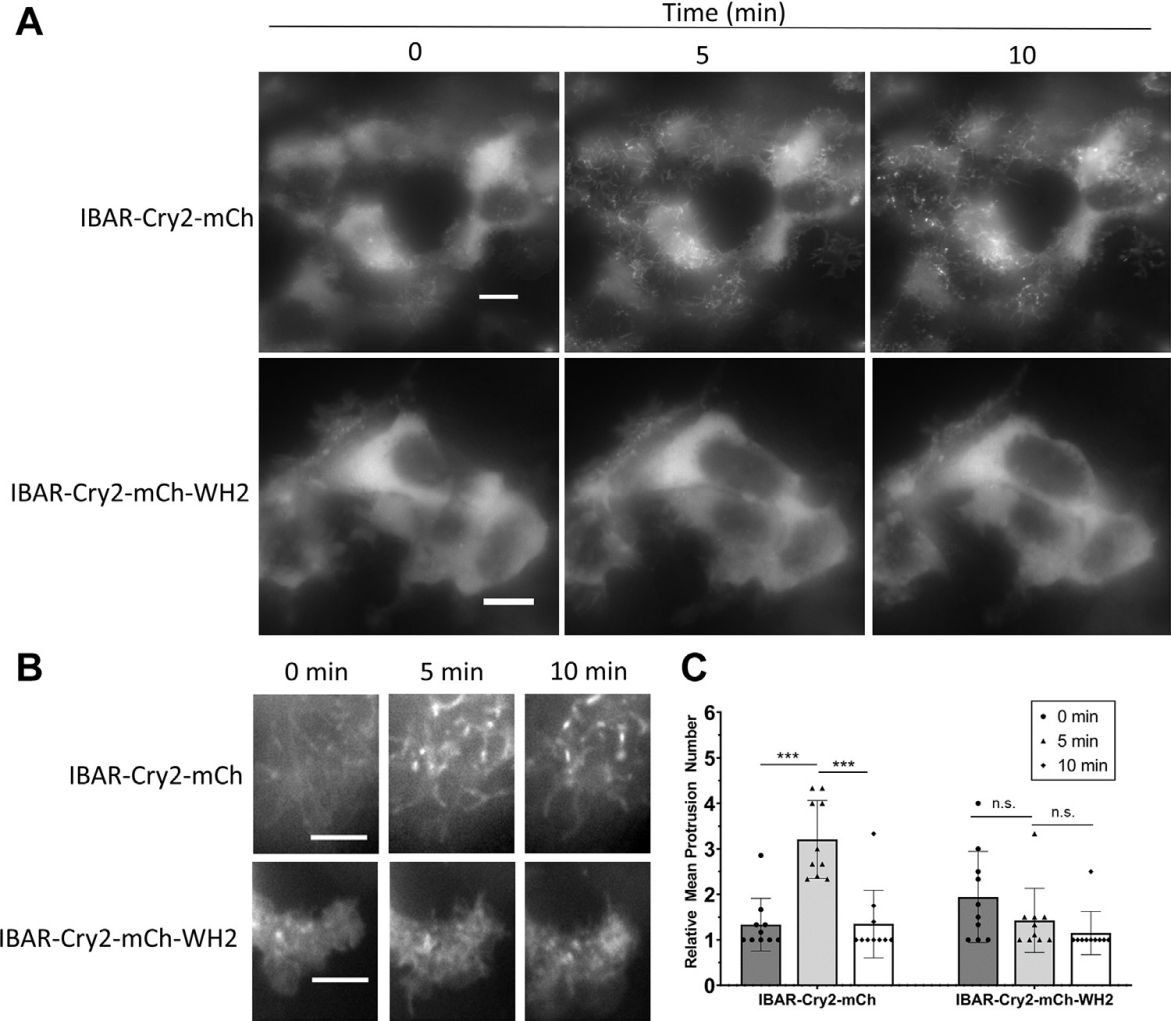
**Light activation of N-terminal I-BAR–Cry2 fusions with and without a C-terminal WH2 domain.***A*, HEK293T cells transfected with I-BAR–Cry2–mCh or I-BAR–Cry2–mCh–WH2 were subjected to blue light illumination (50 ms pulse every 30 s) using a widefield microscope. I-BAR–Cry2–mCh rapidly coalesces into dynamic filopodial structures. The scale bars represent 10 microns. *B*, the presence of a C-terminal WH2 domain results in a cytosolic distribution, reducing impact on filopodial dynamics. The scale bars represent 5 microns. *C*, average number of protrusions at time 0, 5, or 10 min of light exposure from n = 10 cells pooled from three replicate measurements. Statistical analysis conducted with one-way ANOVA (∗∗∗*p* < 0.001; ∗*p* = 0.002; n.s., not significant [*p* > 0.05]). I-BAR labeled as IBAR in figure. BAR, Bin, Amphiphysin, and Rvs; I-BAR, inverse BAR; HEK293T, human embryonic kidney 293T cell line.

Homo-oligomerization of the CRY–BAR constructs I-BAR–Cry2–mCh and Cry2–mCh–I-BAR results in membrane deformation and restriction of cellular dynamics (Movie S1, *A* and *B*). These effects are reversible in the dark. To demonstrate this using Cry2–mCh–I-BAR, we conducted a 10 min light activation sequence, followed by a 30 min observation period in the absence of blue light. Rapid restriction of membrane dynamics was observed during the 10 min blue light activation period, with full reversibility of membrane rounding achieved after 30 min in the absence of blue light (Fig. 4 and Movie S2). We subsequently demonstrated that this effect can be selectively induced using localized illumination of the cell on a confocal microscope (Fig. 5 and Movie S3). We also investigated whether the presence of a WH2 domain (present in I-BAR–Cry2–mCh–WH2 but not in I-BAR–Cry2–mCh and Cry2–mCh–I-BAR) impacted the membrane remodeling capabilities of the optogenetic switch. Light activation of an N-terminal I-BAR domain without a C-terminal WH2 domain (I-BAR–Cry2–mCh) promoted its accumulation in numerous filopodia-like protrusions that rapidly coalesced with continued light exposure (Fig. 6*A*). By contrast, a construct that paired an N-terminal I-BAR domain with a C-terminal WH2-containing domain (I-BAR–Cry2–mCh–WH2) did not accumulate in filopodia-like protrusions (Fig. 6). The interaction between WH2 and monomeric actin and associated complexes (28), which would disfavor the preassociation of I-BAR and PIP2 required for CRY–BAR function, is likely responsible for the observed failure to accumulate at the plasma membrane. Analogously, a recent investigation of the I-BAR protein IRSp53 revealed that the I-BAR domain of IRSp53, expressed as a fusion to GFP, localized to filopodia, whereas the C terminus of IRSp53 was entirely cytosolic (29). Taken together, these results illustrate the modular nature of I-BAR proteins, where the N-terminal I-BAR-containing domain is required for membrane binding and localization, and the WH2 domain is required for recruitment of actin-polymerizing components that promote protrusion formation and elongation (17). Only upon expression as isolated entities are the competing contributions of these domains to subcellular localization apparent. Finally, a side-by-side comparison of light activation of I-BAR–Cry2–mCh, I-BAR–Cry2–mCh–WH2, and Cry2–mCh–I-BAR demonstrated that while robust membrane recruitment is associated with light activation of Cry2–mCh–I-BAR and I-BAR–Cry2–mCh (Movie S4), Cry2–mCh–I-BAR induces a more robust effect on cell membranes, as evidenced by impact on total cell area (Fig. 4*B*). We attribute this to enhanced PIP2 binding and clustering capability of the I-BAR domain when in the C-terminal position of the optogenetic fusion protein.

**Figure 7:**
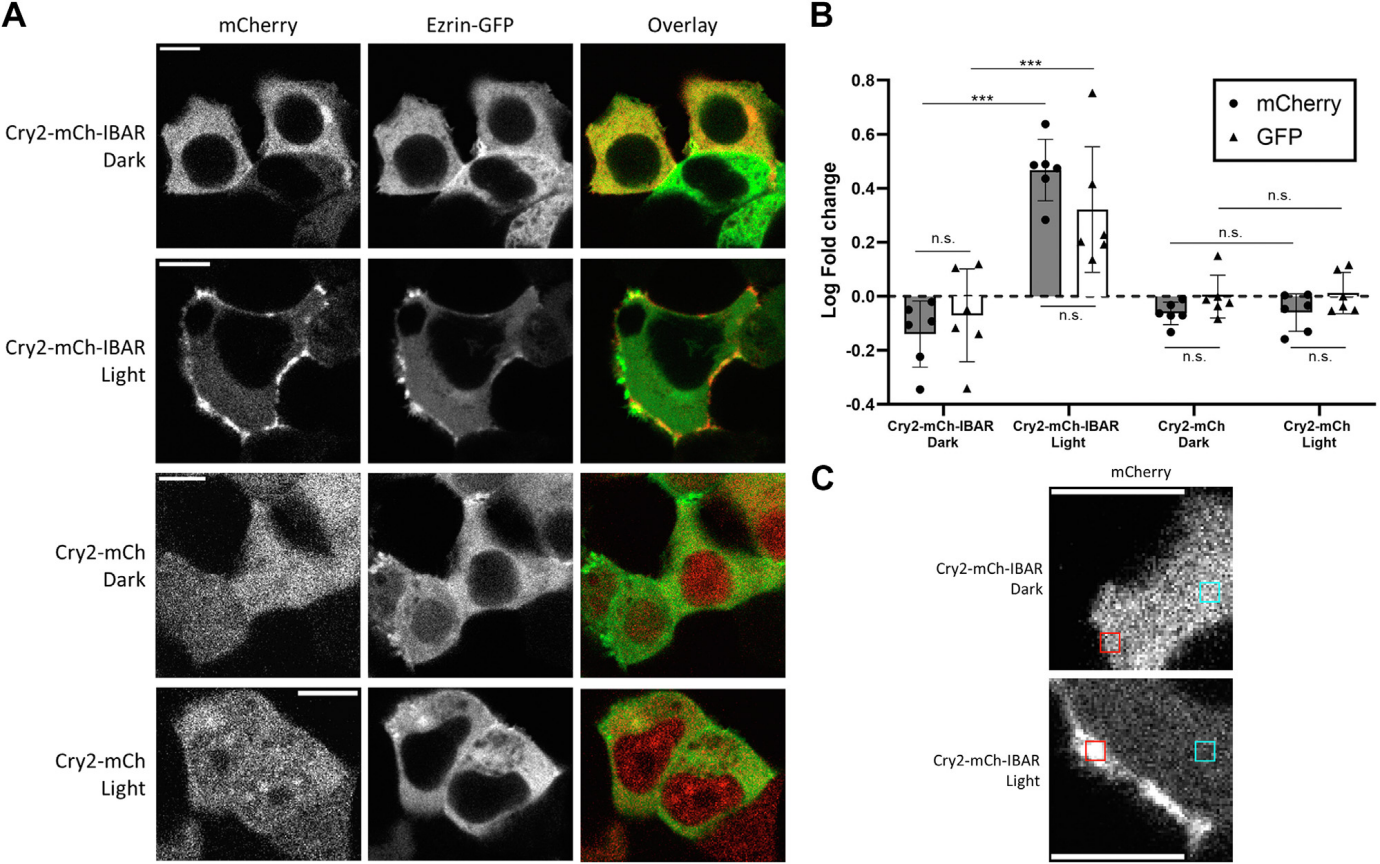
**CRY–BAR activation linked to ezrin.***A*, HEK293T cells cotransfected with Cry2–mCh–I-BAR and ezrin–GFP were subjected to blue light illumination or dark followed by fixation. Confocal microscopy reveals colocalization of CRY–BAR and ezrin–GFP as a result of CRY–BAR activation. The scale bar represents 10 microns. *B*, plot of the log ratio of membrane ROI *versus* cytosol ROI intensity (n = 6 cells per construct), normalized intensity for each channel. ROI dimensions: 1.4 × 1.4 microns. Statistical analysis conducted with one-way ANOVA (∗∗∗*p* < 0.001; n.s., not significant [*p* > 0.05]). *C*, representative images of ROIs positioned in the cytosol (*cyan*) and at the membrane (*red*). The scale bar represents 10 microns. I-BAR labeled as IBAR in figure. BAR, Bin, Amphiphysin, and Rvs; HEK293T, human embryonic kidney 293T cell line; I-BAR, inverse BAR; ROI, region of interest.

**Figure 8:**
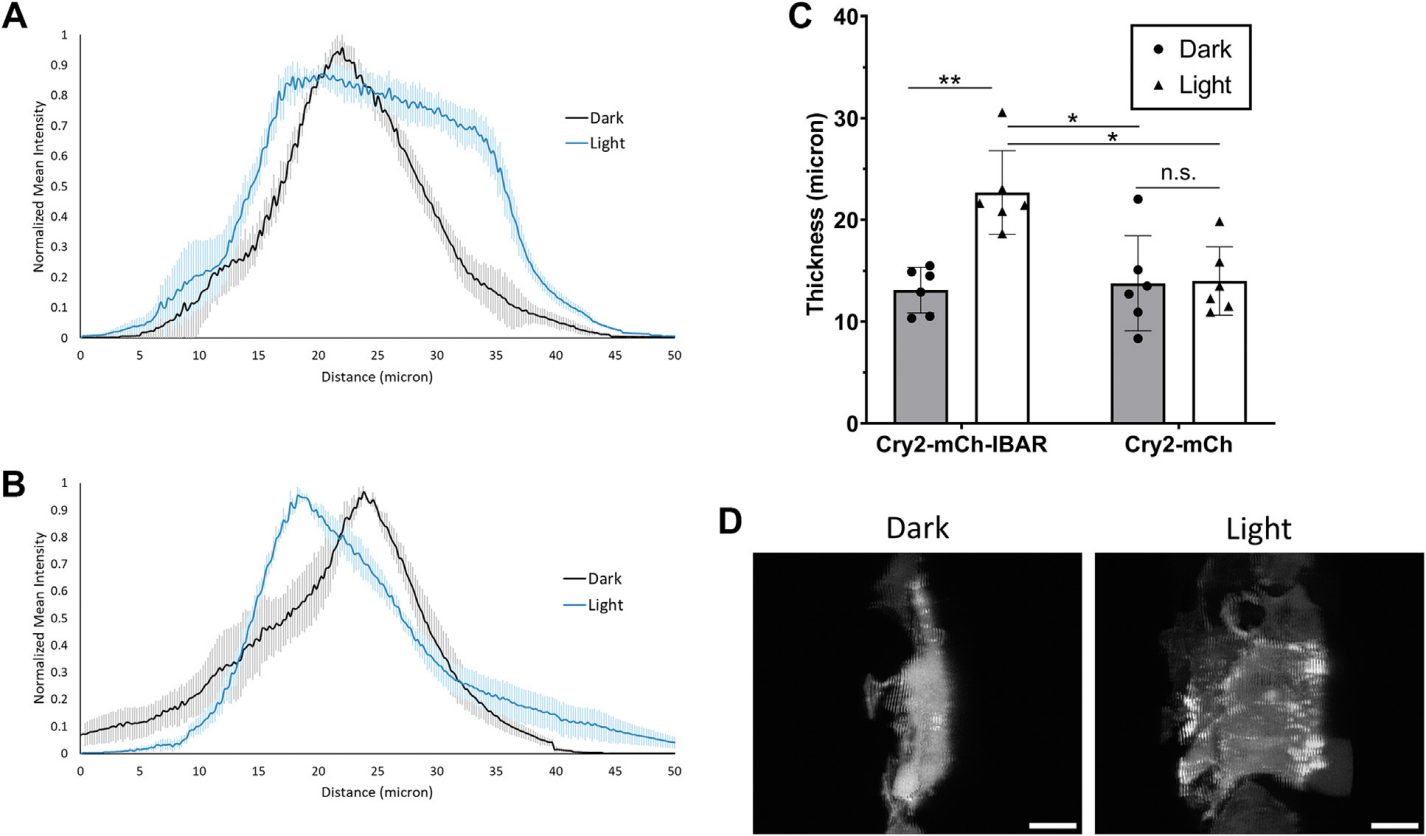
**CRY–BAR activation and cell thickness.** HEK293T cells cotransfected with Cry2–mCh–I-BAR and ezrin–GFP were subjected to blue light illumination or dark followed by fixation. Cellular thickness was analyzed for n = 6 cells per experimental condition. *A*, average cellular thickness of Cry2–mCherry–I-BAR–transfected HEK293T cells before and after illumination. *B*, average cellular thickness of Cry2–mCh–transfected HEK293T cells before and after illumination. *C*, analysis of average cellular thickness at half height (one-way ANOVA: ∗∗*p* = 0.001; ∗*p* = 0.002, n.s., not significant [*p* > 0.05]). *D*, representative images of Z-projections of ezrin–GFP distribution in Cry2–mCherry–I-BAR transfected cells before and after illumination and fixation. The scale bars represent 10 microns. I-BAR labeled as IBAR in figure. BAR, Bin, Amphiphysin, and Rvs; HEK293T, human embryonic kidney 293T cell line; I-BAR, inverse BAR.

**Figure 9:**
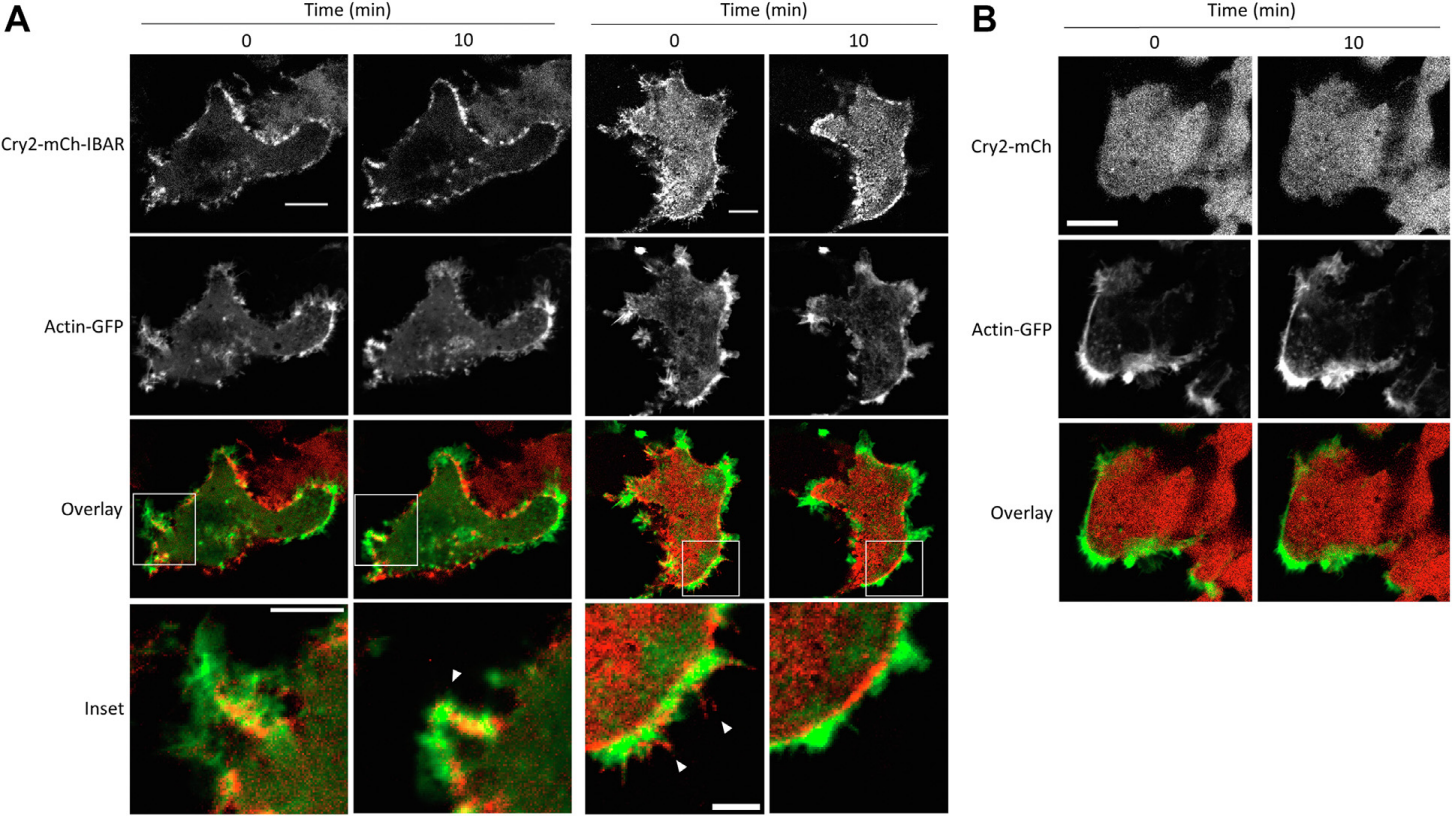
**Coalescence of actin-rich protrusions in response to CRY–BAR activation.***A*, HEK293T cells cotransfected with Cry2–mCh–I-BAR and actin–GFP were illuminated with 488 nm light (5% power) for 10 min on a confocal microscope. Individual images of channels for mCherry (*top row*) and GFP (*second row*) in addition to an overlay (mCherry in *red*; GFP in *green*; inset in *white box*) showed a coalescence of actin-enriched protrusions in conjunction with CRY–BAR-induced membrane rounding after 10 min light exposure (*arrow* in *left cell inset*). Cry2–mCh–I-BAR is also enriched at the tips of actin-enriched protrusions in images before light illumination (*arrows* in *right cell inset*). After 10 min of light exposure, Cry2–mCh–I-BAR is retracted into the cell and no longer observed in protrusions (*right cell inset*, fourth column). Full cell image scale bar represents 10 microns. *Inset image* scale bar represents 5 microns. *B*, HEK293T cells cotransfected with Cry2–mCh and actin–GFP were illuminated with 488 nm light (5% power) for 10 min on a confocal microscope. Individual images of channels for mCherry (*top row*) and GFP (*second row*) in addition to an overlay (mCherry in *red*; GFP in *green*). Cry2–mCh and actin–GFP exhibit no change after 10 min light exposure. The scale bar represents 10 microns. I-BAR labeled as IBAR in figure. BAR, Bin, Amphiphysin, and Rvs; HEK293T, human embryonic kidney 293T cell line; I-BAR, inverse BAR.

We postulated that the dynamic membrane-remodeling activity observed with CRY–BARs might be due to its interaction with proteins that link the plasma membrane with the cytoskeleton. Ezrin is one such protein that has both lipid-binding and cytoskeleton-binding domains (30). We hypothesized that clustering of the PIP2-bound CRY–BARs might impact dynamics by also clustering ezrin, resulting in restriction of cytoskeletal-associated phenomenon such as membrane ruffling. To investigate this, we cotransfected Cry2–mCh–I-BAR with an ezrin–GFP fusion. In the presence of light, we observed colocalization of ezrin–GFP with Cry2–mCh–I-BAR clusters at the plasma membrane (Fig. 7). This effect was pronounced in the light, and absent in the dark, indicating that CRY–BAR activation actively restricts ezrin dynamics. CRY–BAR activation accompanied by ezrin sequestration also resulted in increased cell thickness (Fig. 8), with no such effect being observed in cells expressing the Cry2–mCh control. Accompanying live-cell experiments demonstrate that, while not exclusively so, the accumulation of ezrin near the cell membrane largely followed that of CRY–BAR during light activation and exhibits a close temporal relationship with CRY–BAR activation (Fig. S2). The CRY–BAR–ezrin link implies that CRY–BAR impacts actin dynamics as well. To explore this, we performed live-cell imaging of CRY–BAR light activation in the presence of a GFP-labeled actin (Fig. 9). Actin-rich protrusions were observed to coalesce upon CRY–BAR activation (Fig. 9*A*) but were not generally colocalized with CRY–BAR. Interestingly, in the dark, CRY–BAR is present in filopodia tips and at lamellipodial edges and interspersed with actin (Fig. 9*A*). However, upon light activation, their spatial relationship changes, as rounded CRY–BAR clusters and actin-rich regions at the cell membrane become distinct (Fig. 9*A*, *inset* and Movie S5).

**Figure 10:**
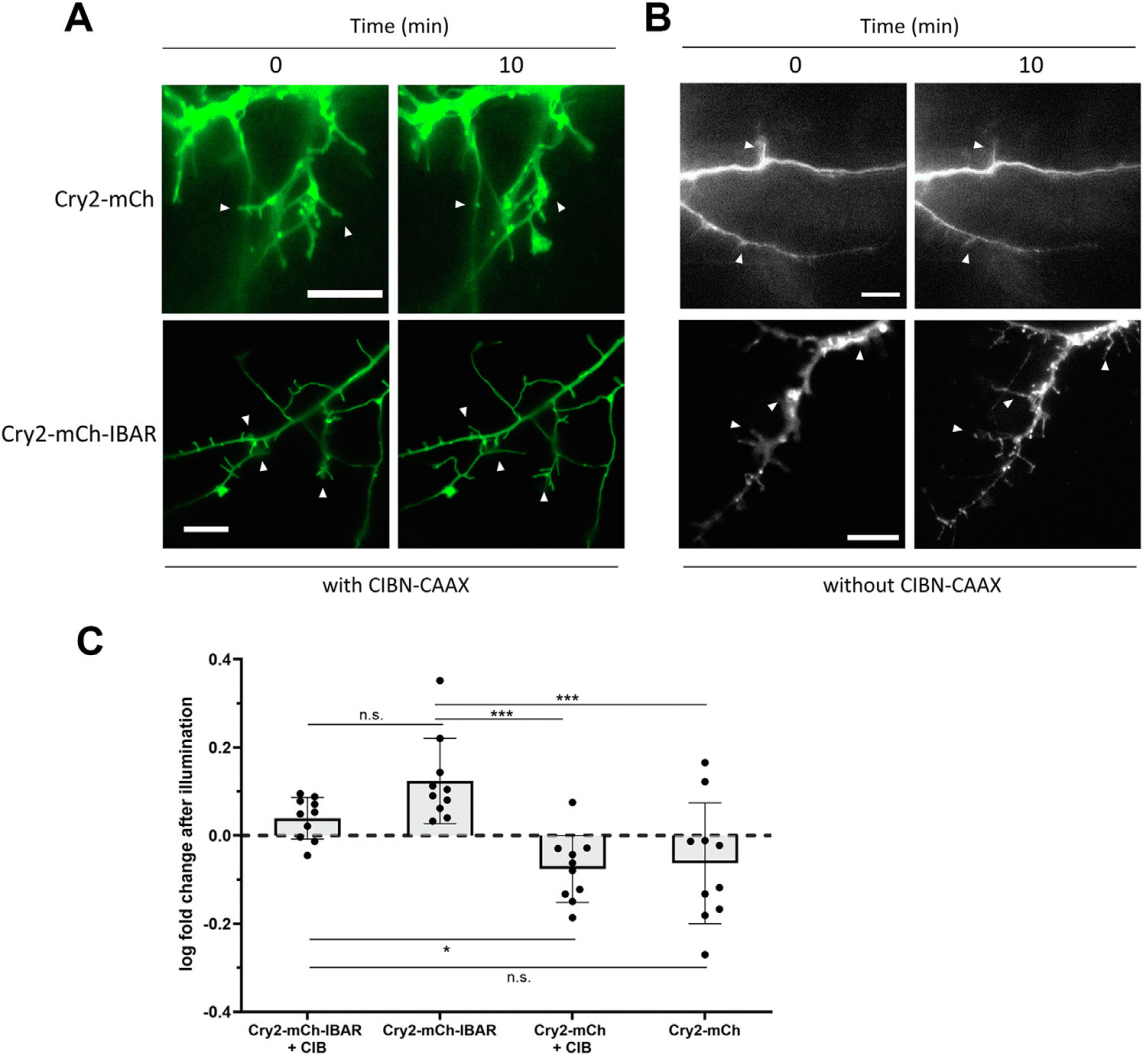
**CRY–BAR plasma membrane recruitment in neurons.** Pre- and 10 min-post 480 nm light illumination of processes in dissociated hippocampal neuron cultures. *A*, neurons cotransfected with Cry2–mCh–I-BAR or Cry2–mCh and CIBN–GFP–CAAX (GFP channel shown). *B*, neurons transfected with Cry2–mCh–I-BAR or Cry2–mCh. *White arrows* indicate example protrusions. The scale bars represent 10 microns. *C*, log fold increase in length of protrusions of Cry2–mCh–I-BAR and Cry2–mCh expressing neurons with or without CIBN–GFP–CAAX (CIB) after 10 min of 480 nm light illumination (n = 10 neurons per construct; neurons from three separate cultures; number of protrusions per neuron analyzed: Cry2–mCh–I-BAR + CIB: range 24–141, average 65; Cry2–mCh–I-BAR: range 35–75, average 50; Cry2–mCh + CIB: range 34–116, average 61; Cry2–mCh: range 4–17; average 10. Statistical analysis conducted with one-way ANOVA: ∗∗∗*p* < 0.001; ∗*p* = 0.04; n.s.: *p* > 0.05). Error bars represent standard deviations. I-BAR labeled as IBAR in figure. BAR, Bin, Amphiphysin, and Rvs; I-BAR, inverse BAR; n.s., not significant.

Having investigated the CRY–BAR response in immortalized cell culture, we next evaluated the capability of the sensor to report membrane expansion dynamics in neurons. For this, dissociated postnatal cortical neuron cultures prepared from newborn mice were transfected with Cry2–mCh–I-BAR, or cotransfected with CIBN–GFP–CAAX, according to protocol we developed for expressing optogenetic sensors in primary cultures (31). Light activation of Cry2–mCh–I-BAR, but not Cry2–mCh, resulted in elongation of neuronal processes that might indicate early stage spinogenesis (Fig. 10, Movies S6 and S7). There was no apparent benefit to activation of Cry2–mCh–I-BAR in the presence of CIBN–GFP–CAAX, providing additional evidence that activation of Cry2–mCh–I-BAR alone is sufficient for effecting membrane dynamics (Fig. 10*C*). Accordingly, whole-cell illumination of neurons transfected with only Cry2–mCh–I-BAR results in abundant membranous cluster formation throughout neuronal processes (Fig. S3*A*). Notably, oligomerization of I-BAR domain proteins is essential for their membrane-remodeling activities (32). In the context of CRY–BAR, optimal membrane remodeling is achieved *via* the homo-oligomerization of Cry2. By contrast, recruitment of CRY–BAR to CIBN–GFP–CAAX, while not completely detrimental to CRY–BAR activity, results in less membrane activity than activation of CRY–BAR alone (Fig. 10*C*). This is presumably because of a combination of reduced CRY2 homo-oligomerization in the presence of CIB, a phenomenon that has been previously described (22), and possible orientation effects of the Cry2–CIB interaction on the I-BAR domain. CIBN–GFP–CAAX has a similar membrane distribution to CRY–BAR and could interfere with CRY–BAR activity at the cell membrane (Fig. S4).

**Figure 11:**
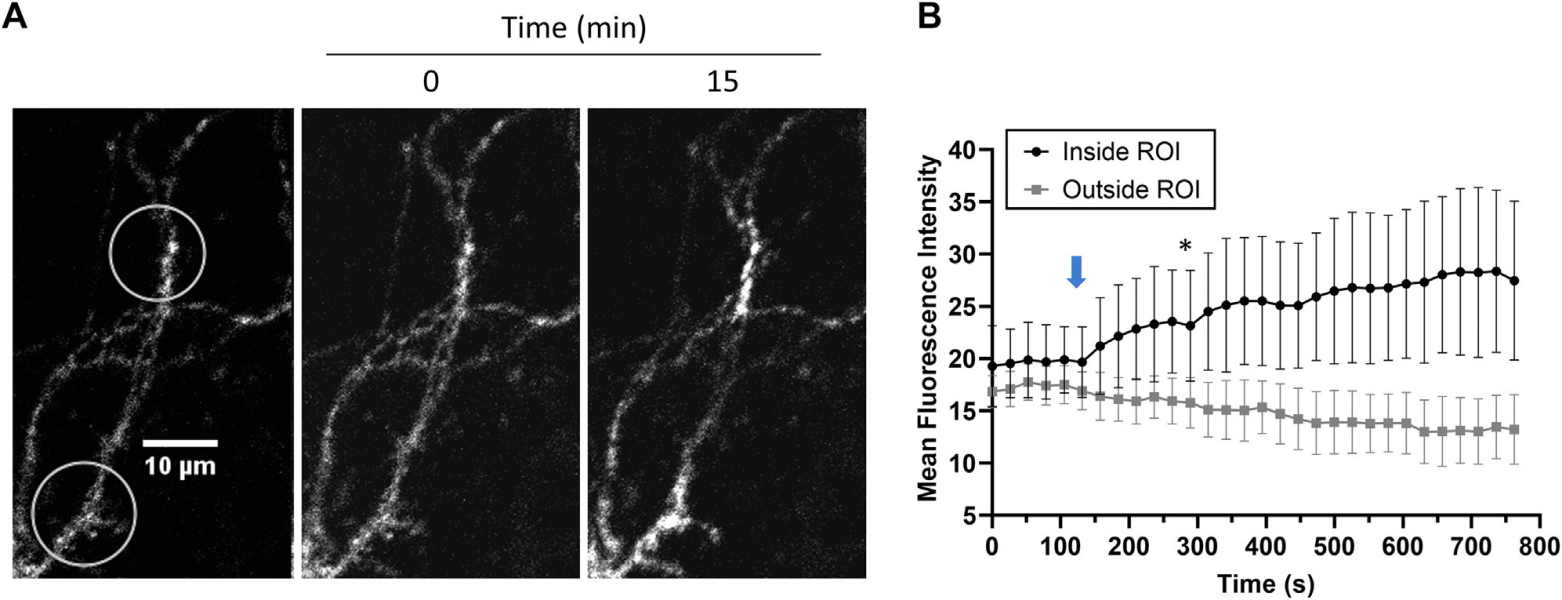
**Localized light activation of CRY–BAR in neurons.***A*, neurons transfected with Cry2–mCh–I-BAR were subjected to restricted blue light illumination (*white circles*) using a confocal microscope. Clustering of CRY–BAR is apparent in the areas illuminated 15 min post–blue light stimulation (irradiation with 488 nm laser at 5% power at the 120 s mark). The scale bar represents 10 microns. *B*, a plot of fluorescence intensity from four illuminated ROIs *versus* four nonilluminated regions is shown at *right* (error bars = standard deviation; differences are statistically significant [one-way ANOVA, *p* < 0.05] beginning at the 263 s time point [∗] and beyond). *Blue arrow* indicates time of blue light illumination. BAR, Bin, Amphiphysin, and Rvs; I-BAR, inverse BAR; ROI, region of interest.

Finally, using localized illumination conditions, we demonstrated that Cry2–mCh–I-BAR can be selectively activated in neuronal processes (Fig. 11 and Movie S8). Taken together, this work provides evidence that the Cry2–mCh–I-BAR molecular optogenetic tool is suitable for the targeted manipulation of cytoskeletal structures and dynamics at the plasma membrane. To our knowledge, this switch represents the first application of the I-BAR domain from MTSS1 in an optogenetic switch. In comparison to prior work in this area, CRY–BAR has the advantage of being a single-component switch, which can reduce experimental complexity and facilitate its implementation into transgenic model organisms. We note that the mechanisms of I-BAR domain proteins are not completely understood, and there are structural and mechanistic differences in the I-BAR domains across different proteins (26). As a result, one can reasonably anticipate that the incorporation of I-BAR domains from different I-BAR-containing proteins and their orthologs could produce different experimental phenotypes within the same optogenetic background. This intriguing possibility illustrates the value of pursuing multiple optogenetic variations of BAR domain proteins. Potential applications of CRY–BARs include site-directed mutagenesis of the I-BAR domain, which could provide valuable structure–function information regarding the mechanism of the I-BAR–plasma membrane interaction, and investigation of membrane deformation–dependent cell signaling pathways, such as calcium signaling (33).

## Conclusion

In this report, we describe the development of a family of optogenetic switches, collectively named CRY–BAR, that comprise a versatile platform for controlling membrane dynamics in live cells with high spatial and temporal resolution. These switches combine homo- and hetero-oligomerization of the Cry2–CIB photoreceptor system with the innate PIP2-binding affinity of the I-BAR domain. As their function varies depending on the presence of various functional elements, such as WH2-binding domains, we anticipate that a modular approach can be undertaken to adapt these optogenetic switches for other applications, including the recruitment of enzyme and receptor-activating and -inhibitory domains. Finally, CRY–BARs are suitable tools to study membrane dynamics not only in immortalized cells but also in sensitive and difficult to transfect primary cultures, such as neurons. Therefore, the CRY–BAR optogenetic switches we report here are expected to have wide applicability for investigating cellular processes associated with membrane dynamics in a variety of experimental paradigms.

## Experimental procedures

### Plasmids and cloning

Cloning of I-BAR–Cry2–mCh, Cry2–mCh–I-BAR, I-BAR–Cry2–mCh–WH2, and Cry2–mCh–MTSS1 constructs was conducted using a previously described cloning scheme (34). Briefly, the I-BAR domain from MTSS1, the WH2 domain from MTSS1, and MTSS1 were PCR amplified from human MTSS1 complementary DNA obtained from the Arizona State University DNA repository (DNASU ID: HsCD00746054). N-terminal I-BAR genes were cloned into Cry2PHR–mCherry (Addgene; #26866) using NheI and XhoI restriction sites (23). C-terminal I-BAR, WH2, and MTSS1 genes were cloned into Cry2PHR–mCherry using BsrgI and NotI restriction sites. The ezrin–GFP expression construct was a gift from Stephen Shaw (plasmid pHJ421; Addgene; #20680 (35)). The actin expression construct (pCAG-mGFP-actin; Addgene; #21948) was a gift from Ryohei Yasuda. CIBN–GFP–CAAX (pCIBN(deltaNLS)-pmGFP; Addgene; #26867) was a gift from Chandra Tucker. Midi prep quantities of DNA of each construct were created from *Escherichia coli* and collected for cell transfection.

### Cell lines and transfection

HEK293T cells (passage 8) used for these experiments were purchased from American Type Culture Collection and were cultured in Dulbecco's modified Eagle's medium (DMEM) containing 10% fetal bovine serum (FBS) and 1% penicillin–streptomycin. Cultures were transfected at 70% confluency with the Lipofectamine 3000 reagent (Invitrogen) following the manufacturer’s suggested protocols. Briefly, for dual transfections in 35 mm glass bottom dishes for cell imaging or 6-well culture plates for lysis, plasmid DNA was combined in a 1:1 ratio (1250 ng per plasmid) in 125 μl of Opti-MEM, followed by the addition of 5 μl of P3000 reagent. For single transfections, 2500 ng of plasmid DNA was used per transfection. In a separate vial, 3.75 μl of Lipofectamine 3000 were added to 125 μl of Opti-MEM. The two 125 μl solutions were combined and allowed to incubate at room temperature for 10 min, followed by dropwise addition to cell culture. For ezrin–GFP and actin–GFP cotransfection with CRY–BAR, 2000 ng of CRY–BAR DNA and 1000 ng of GFP DNA were added to 100 μl of DMEM along with subsequent addition of 3 μl of CalFectin (SignaGen Laboratories). After 10 min of incubation at room temperature, the solution was added dropwise to cell cultures. Each transfection solution remained on cells overnight. Transfected cells were maintained at 37 °C and 5% CO_2_ in a humidified tissue culture incubator, in culture medium consisting of DMEM supplemented with 10% FBS and 1% penicillin–streptomycin.

### Neuron cultures and transfection

Postnatal-dissociated cortical neuron cultures were prepared as previously described (31, 36), from newborn B6 mice. Neurons were plated into 35 mm glass bottom Petri dishes at a 1 million/ml density in culture medium consisting of Basal Medium Eagle supplemented with 10% bovine calf serum and 1% penicillin–streptomycin. On day *in vitro* 2 (DIV2), culture medium was changed to Neurobasal A medium, supplemented with B27-plus reagent (Invitrogen), Glutamax, and 1% penicillin–streptomycin. Neurons were transfected with the CryBAR optogenetic system (6 μg plasmid/plate) on DIV5 using Lipofectamine LTX reagent. On DIV7, culture medium was removed and neurons were placed in imaging solution (Mg-free HEPES-buffered artificial cerebrospinal fluid (37)) Live-cell imaging was performed before and after illumination using a Leica DMi8 Live Cell Imaging System. Animal use protocols were approved by East Carolina University Institutional Animal Care and Use Committee.

### Fixed and live-cell imaging preparation

#### Fixed cell experiments

Immediately prior to fixation, transfected HEK293T cells were either kept in dark conditions or continually illuminated with LED blue light (Sunlite LED Par30 Reflector, item #80021, 4 W, 120 V, 66.21 μmol/s/m^2^; placed 10 cm from cell culture dishes) for 5 min. Cells were washed with Dulbecco’s PBS (DPBS) (with calcium and magnesium; 1× 1 ml), then fixed for 10 min at room temperature with prewarmed 4% paraformaldehyde solution in DPBS (37 °C; prepared from 16% paraformaldehyde [Electron Microscopy Sciences]). Following fixation, cells were washed with DPBS and then stored in DPBS at 4 °C.

#### Live-cell experiments

Transfected HEK293T cell media were replaced with 10% FBS in Leibovitz's L-15 medium. Cells were allowed to equilibrate in the live-cell incubation system (OKOLab) for 10 min prior to beginning the illumination sequence.

### Imaging

#### Confocal microscopy

Confocal images of fixed cells were collected with a Zeiss LSM 700 laser scanning microscope using ZEN Black 2012 software. Ezrin–GFP and actin–GFP live-cell images were collected with a Zeiss LSM 700 laser scanning microscope equipped with a stage-top incubator and ZEN Black 2012 software. Fluorescence images were colorized and overlaid using FIJI software (38) equipped with Bio Formats (39).

#### Widefield microscopy

A Leica DMi8 Live Cell Imaging System, equipped with an OKOLab stage-top live cell incubation system, LASX software, Leica HCX PL APO 63×/1.40 to 0.60 numerical objective oil objective, Lumencor LED light engine, CTRadvanced+ power supply, and a Leica DFC900 GT camera, was used to acquire images. Exposure times were set at 200 ms (mCherry, 553 nm) and 50 ms (GFP, 480 nm), with LED light sources at 50% power, and images were acquired every 30 s over specified time course.

### Western blotting

Transfected HEK293T cells were lysed with 200 μl of M-PER lysis buffer (Thermo Fisher Scientific) containing 1× Halt protease–phosphatase inhibitor cocktail (Thermo Fisher Scientific). After 10 min on ice, lysates were collected and centrifuged for 15 min (94 rcf; 4 °C). The supernatants were combined with Laemmli SDS sample buffer (Alfa Aesar) and incubated at 65 °C for 10 min. The resulting lysates were subjected to electrophoresis on a 10% SDS-PAGE gel and then transferred onto polyvinylidene fluoride membranes (20 V, 800 min). Membranes were then blocked for 1 h with 5% bovine serum albumin (BSA) in 1× Tris-buffered saline (TBS) with 1% Tween (TBST), followed by incubation with primary antibody (anti-mCherry antibody [Cell Signaling] 1:1000 dilution in 5% BSA–TBST; anti-GAPDH antibody [Invitrogen] 1:1000 dilution in 5% BSA–TBST) overnight at 4 °C on a platform rocker. The membranes were then washed 3× for 5 min each with TBST and incubated with the appropriate secondary antibody in 5% BSA–TBST (1 h; room temperature). After washing 3× for 5 min with TBST, the membranes were exposed to a chemiluminescent substrate for 5 min and imaged with an Azure cSeries imaging station.

### Statistical analyses

Statistical significance (*p* values; Figure 4, Figure 5, Figure 6, Figure 7, Figure 8, Figure 10, Figure 11) was determined using a one-way ANOVA (Holm–Sidak method) in SigmaPlot v. 13.0 (Systat Software, Inc). Mean ± S.D. plots were constructed using GraphPad Prism version 8.2.1 for Windows, GraphPad Software, San Diego, California, USA (www.graphpad.com).

## Data availability

Experimental data are available upon request. All CRY–BAR plasmids will be available through Addgene, a nonprofit DNA repository.

## Supporting information

This article contains supporting information.

## Conflict of interest

The authors declare that they have no conflicts of interest with the contents of this article.

## Supporting information

Supplemental Movie S1A **Fluorescence widefield microscopy of Cry2-mCh-IBAR light activation in HEK293T cells.** Whole cell activation of Cry2-mCh-IBAR results in filopodial clustering and retraction. Images acquired every 30 s (553 nm; 200 ms exposure). Blue light activation (470 nm, 50 ms pulse) protocol: 0:00 to 5:30 min:s (no blue light); 6:00 to 16:30 min:s (blue light pulse every 30 s); 17:00 – end min:s (no blue light)

Supplemental Movie S1B **Fluorescence widefield microscopy of Cry2-mCh-IBAR light activation in HEK293T cells; this movie shows alternate focal plane to highlight filopodial localization.** Whole cell activation of Cry2-mCh-IBAR results in filopodial clustering and retraction. Images acquired every 30 s (553 nm; 200 ms exposure). Blue light activation (470 nm, 50 ms pulse) protocol: 0:00 to 4:30 min:s (no blue light); 5:00 to 19:30 min:s (blue light pulse every 30 s); 17:00 – end min:s (no blue light)

Supplemental Movie S2 **Fluorescence widefield microscopy of Cry2-mCh-IBAR light activation in HEK293T cells; zoomed in region to show clustering at cell edge in detail and reversibility over a longer dark recovery timecourse (30 min) than shown in**Movie S1***A*.** Whole cell activation of Cry2-mCh-IBAR results in filopodial clustering and retraction. Images acquired every 30 s (553 nm; 200 ms exposure). Blue light activation (470 nm, 50 ms pulse) protocol: 0:00 to 5:00 min:s (no blue light); 5:30 to 16:00 min:s (blue light pulse every 30 s); 16:30 to 46:00 min:s (no blue light)

Supplemental Movie S3 **Localized Cry2-mCh-IBAR light activation in HEK293T cells with confocal microscope.** Three ROIs (shown at beginning of movie) subjected to selective activation with 488 nm laser (5% power). Light pulse applied at 4:00 min:s

Supplemental Movie S4 **Side-by-side movies showing fluorescence widefield microscopy of CRY-BAR(s) light activation in HEK293T cells; whole cell activation results in filopodial clustering and retraction in Cry2-mCh-IBAR (100% observed in 51 cells pooled from 8 replicates) and in IBAR-Cry2-mCh (98% observed in 44 cells pooled from 6 replicates); whereas IBAR-Cry2-mCh-WH2 (96% observed in 52 cells pooled from 7 replicates) remains cytosolic during light activation and does not impact filopodia.** Images acquired every 30 s (553 nm; 200 ms exposure). Blue light activation (470 nm, 50 ms pulse) protocol: 0:00 to 5:00 min:s (no blue light); 5:30 to 16:00 min:s (blue light pulse every 30 s); 16:30 to 46:00 min:s (no blue light)

Supplemental Movie S5 **Confocal microscopy of Cry2-mCh-IBAR light activation in presence of Actin-GFP in HEK293T cell.** Simultaneous activation and imaging of GFP with 488 nm laser (5% power) occurs every 30 s. *Rightmost panel* shows overlay of Cry2-mCh-IBAR (*red*) with Actin-GFP (*green*)

Supplemental Movie S6 **Fluorescence widefield microscopy of Cry2-mCh-IBAR/CIBN-GFP-CAAX light activation in neuronal process.** Blue light activation (470 nm, 50 ms pulse) protocol: 0:00 to 5:00 min:s (no blue light); 5:30 – end min:s (blue light pulse every 30 s)

Supplemental Movie S7 **Fluorescence widefield microscopy of Cry2-mCh-IBAR light activation in neuronal process.** Blue light activation (470 nm, 50 ms pulse) protocol: 0:00 to 5:00 min:s (no blue light); 5:30 – end min:s (blue light pulse every 30 s)

Supplemental Movie S8 **Localized Cry2-mCh-IBAR light activation in neuronal process with confocal microscope.** Three ROIs (shown at beginning of movie) subjected to selective activation with 488 nm laser (5% power). Light pulse applied at 4:00 min:s

Supplemental Figure S1 **CRY-BAR construct design and expression.***A*, diagrams of the CRY-BAR protein fusions; Constructs 2, 3, and 4 contain portions of the MTSS-1 protein (I-BAR, WH2, or both); Construct 5 contains the full length MTSS-1 protein. *B*, lysates of HEK293T cells transfected with CRY-BAR constructs and controls (Lanes 1–6, numbers correspond to panel *A*) were Western blotted with anti-mCherry antibody. *Boxes* indicate the apparent molecular weights: 1. 85 kDa; 2. 111 kDa; 3. 130 kDa; 4. 111 kDa; 5. 168 kDa; 6. No transfect control. The Western blot is representative of four independent trials

Supplemental Figure S2 **Temporal relationship of Cry2-mCh-IBAR and Ezrin-GFP localization.***A*, HEK293T cells transfected with Cry2-mCh-IBAR and Ezrin-GFP were illuminated every 30 s with 488 nm light (5% power) on a confocal microscope over 20 min to assess their localization patterns in response to light activation of Cry2. Areas at the membrane exhibiting colocalization of Cry2-mCh-IBAR and Ezrin-GFP were analyzed for intensity over time by conducting a 6 micron line scan (*cyan*) ranging from the cytosol, through the membrane, to outside of the cell. Scale bar = 5 microns. *B*, HEK293T cells transfected with Cry2-mCh and Ezrin-GFP were imaged similarly to (*A*). Co-expressing cells were analyzed for intensity over time using a line scan (*cyan*) as described in (*A*). Line length: 6 microns. Scale bar = 5 microns. *C*, plot of intensity at the membrane (around 3 micron point of line) over the intensity in the cytosol (at the cytosolic end of line) from line analysis shown in (*A*). *D*, plot of intensity at the membrane (around 3 micron point of line) over the intensity in the cytosol (at the cytosolic end of line) from line analysis shown in (*B*). For (*C*) and (*D*), each point of intensity was taken at time 0 min of light exposure and then in 1 min increments for a total of 20 min (n = 6 cells). Membrane to cytosol ratios were normalized for intensity for each time point before averaging (error bars = standard deviation)

Supplemental Figure S3 **Global activation of CRY-BAR in hippocampal neurons.***A*, neurons transfected with Cry2-mCh-IBAR; and co-transfected with Cry2-mCh-IBAR and CIBN-GFP-CAAX were illuminated every 30 s with 470 nm light on a widefield microscope. After 10 min, Cry2-mCh-IBAR forms numerous clusters throughout the neuronal processes, whereas in the presence of CIB, no such clusters are formed. Scale bar = 10 microns. *B*, full view of neurons transfected with Cry2-mCh-IBAR or Cry2-mCh. Neurons were illuminated every 30 s with 470 nm light on a widefield microscope. Scale bar = 10 microns. *C*, full view of neurons co-transfected with CIBN-GFP-CAAX (*right*; *green*) and Cry2-mCh-IBAR or Cry2-mCh (*left*; *grey*). Neurons were illuminated every 30 s with 470 nm light on a widefield microscope. Scale bar = 10 microns

Supplemental Figure S4 **Dark Localization of Cry2-mCh-IBAR and CIB-GFP-CAAX.** HEK293T cells transfected with Cry2-mCh-IBAR and CIBN-GFP-CAAX were imaged sequentially (mCherry followed by GFP) to assess their localization patterns prior to light activation of Cry2. Both constructs have similar patterns of plasma membrane localization. Scale bar = 10 microns

## References

[1] Kichuk T.C., Carrasco-López C., Avalos J.L. (2021). Lights up on organelles: optogenetic tools to control subcellular structure and organization. WIREs Mech. Dis..

[2] Klewer L., Wu Y.-W. (2019). Light-induced dimerization approaches to control cellular processes. Chemistry.

[3] Ueda Y., Sato M. (2018). Cell membrane dynamics induction using optogenetic tools. Biochem. Biophys. Res. Commun..

[4] Khamo J.S., Krishnamurthy V.V., Chen Q., Diao J., Zhang K. (2019). Optogenetic delineation of receptor tyrosine kinase subcircuits in PC12 cell differentiation. Cell Chem. Biol..

[5] O'Banion C.P., Priestman M.A., Hughes R.M., Herring L.E., Capuzzi S.J., Lawrence D.S. (2018). Design and profiling of a subcellular targeted optogenetic cAMP-dependent protein kinase. Cell Chem. Biol..

[6] O'Banion, Colin P., Vickerman B.M., Haar L., Lawrence D.S. (2019). Compartmentalized cAMP generation by engineered photoactivated adenylyl cyclases. Cell Chem. Biol..

[7] Shaaya M., Fauser J., Zhurikhina A., Conage-Pough J.E., Huyot V., Brennan M. (2020). Light-regulated allosteric switch enables temporal and subcellular control of enzyme activity. ELife.

[8] Wu Y.I., Frey D., Lungu O.I., Jaehrig A., Schlichting I., Kuhlman B. (2009). A genetically encoded photoactivatable Rac controls the motility of living cells. Nature.

[9] Wu Y.I., Wang X., He L., Montell D., Hahn K.M. (2011). Spatiotemporal control of small GTPases with light using the LOV domain. Methods Enzymol..

[10] Redchuk T.A., Karasev M.M., Verkhusha P.V., Donnelly S.K., Hülsemann M., Virtanen J. (2020). Optogenetic regulation of endogenous proteins. Nat. Commun..

[11] Hughes J.H., Kumar S. (2016). Synthetic mechanobiology: engineering cellular force generation and signaling. Curr. Opin. Biotechnol..

[12] Antonny B. (2011). Mechanisms of membrane curvature sensing. Annu. Rev. Biochem..

[13] Linkner J., Witte G., Zhao H., Junemann A., Nordholz B., Runge-Wollmann P. (2014). The inverse BAR domain protein IBARa drives membrane remodeling to control osmoregulation, phagocytosis and cytokinesis. J. Cell Sci..

[14] Prévost C., Zhao H., Manzi J., Lemichez E., Lappalainen P., Callan-Jones A. (2015). IRSp53 senses negative membrane curvature and phase separates along membrane tubules. Nat. Commun..

[15] Pykäläinen A., Boczkowska M., Zhao H., Saarikangas J., Rebowski G., Jansen M. (2011). Pinkbar is an epithelial-specific BAR domain protein that generates planar membrane structures. Nat. Struct. Mol. Biol..

[16] Saarikangas J., Kourdougli N., Senju Y., Chazal G., Segerstråle M., Minkeviciene R. (2015). MIM-induced membrane bending promotes dendritic spine initiation. Dev. Cell.

[17] Saarikangas J., Mattila P.K., Varjosalo M., Bovellan M., Hakanen J., Calzada-Wack J. (2011). Missing-in-metastasis MIM/MTSS1 promotes actin assembly at intercellular junctions and is required for integrity of kidney epithelia. J. Cell Sci..

[18] Yu D., Zhan X.H., Niu S., Mikhailenko I., Strickland D.K., Zhu J. (2011). Murine missing in metastasis (MIM) mediates cell polarity and regulates the motility response to growth factors. PLoS One.

[19] Zhao H., Pykäläinen A., Lappalainen P. (2011). I-BAR domain proteins: linking actin and plasma membrane dynamics. Curr. Opin. Cell Biol..

[20] Jones T., Liu A., Cui B. (2020). Light-inducible generation of membrane curvature in live cells with engineered BAR domain proteins. ACS Synth. Biol..

[21] Li L., Liu H., Baxter S.S., Gu N., Ji M., Zhan X. (2016). The SH3 domain distinguishes the role of I-BAR proteins IRTKS and MIM in chemotactic response to serum. Biochem. Biophys. Res. Commun..

[22] Che D.L., Duan L., Zhang K., Cui B. (2015). The dual characteristics of light-induced Cryptochrome 2, homo-oligomerization and heterodimerization, for optogenetic manipulation in mammalian cells. ACS Synth. Biol..

[23] Kennedy M.J., Hughes R.M., Peteya L.A., Schwartz J.W., Ehlers M.D., Tucker C.L. (2010). Rapid blue-light-mediated induction of protein interactions in living cells. Nat. Methods.

[24] Park H., Kim N.Y., Lee S., Kim N., Kim J., Heo W.D. (2017). Optogenetic protein clustering through fluorescent protein tagging and extension of CRY2. Nat. Commun..

[25] Taslimi A., Vrana J.D., Chen D., Borinskaya S., Mayer B.J., Kennedy M.J. (2014). An optimized optogenetic clustering tool for probing protein interaction and function. Nat. Commun..

[26] Saarikangas J., Zhao H., Pykäläinen A., Laurinmäki P., Mattila P.K., Kinnunen P.K.J. (2009). Molecular mechanisms of membrane deformation by I-BAR domain proteins. Curr. Biol..

[27] Chatzi C., Zhang Y., Hendricks W.D., Chen Y., Schnell E., Goodman R.H. (2019). Exercise-induced enhancement of synaptic function triggered by the inverse BAR protein, Mtss1L. Elife.

[28] Paunola E., Mattila P.K., Lappalainen P. (2002). WH2 domain: a small, versatile adapter for actin monomers. FEBS Lett..

[29] Pipathsouk A., Brunetti R.M., Town J.P., Graziano B.R., Breuer A., Pellett P.A. (2021). The WAVE complex associates with sites of saddle membrane curvature. J. Cell Biol..

[30] Tsai F.-C., Bertin A., Bousquet H., Manzi J., Senju Y., Tsai M.-C. (2018). Ezrin enrichment on curved membranes requires a specific conformation or interaction with a curvature-sensitive partner. Elife.

[31] Bunner W.P., Dodson R., Szatmari E.M., Hughes R.M. (2021). Transfection and activation of CofActor, a light and stress gated optogenetic tool, in primary hippocampal neuron cultures. Bio Protoc..

[32] Nepal B., Sepehri A., Lazaridis T. (2021). Mechanism of negative membrane curvature generation by I-BAR domains. Structure.

[33] Tu M.K., Levin J.B., Hamilton A.M., Borodinsky L.N. (2016). Calcium signaling in skeletal muscle development, maintenance and regeneration. Cell Calcium.

[34] Salem F.B., Bunner W.P., Prabhu V.V., Kuyateh A.B., O'Bryant C.T., Murashov A.K. (2020). CofActor: a light- and stress-gated optogenetic clustering tool to study disease-associated cytoskeletal dynamics in living cells. J. Biol. Chem..

[35] Hao J.-J., Liu Y., Kruhlak M., Debell K.E., Rellahan B.L., Shaw S. (2009). Phospholipase C-mediated hydrolysis of PIP2 releases ERM proteins from lymphocyte membrane. J. Cell Biol..

[36] Chang J.-Y., Parra-Bueno P., Laviv T., Szatmari E.M., Lee S.-J.R., Yasuda R. (2017). CaMKII autophosphorylation is necessary for optimal integration of Ca(2+) signals during LTP induction, but not maintenance. Neuron.

[37] Sun Y., Smirnov M., Kamasawa N., Yasuda R. (2021). Rapid Ultrastructural changes in the PSD and surrounding membrane after induction of structural LTP in single dendritic spines. J. Neurosci..

[38] Schindelin J., Arganda-Carreras I., Frise E., Kaynig V., Longair M., Pietzsch T. (2012). Fiji: an open-source platform for biological-image analysis. Nat. Methods.

[39] Linkert M., Rueden C.T., Allan C., Burel J.-M., Moore W., Patterson A. (2010). Metadata matters: access to image data in the real world. J. Cell Biol..

[40] Cao M., Zhan T., Ji M., Zhan X. (2012). Dimerization is necessary for MIM-mediated membrane deformation and endocytosis. Biochem. J..

