# Supplementary figures and images for: CRY–BARs: Versatile light-gated molecular tools for the remodeling of membrane architectures
Source: J Biol Chem. 2022 Aug 18;298(10):102388. doi: 10.1016/j.jbc.2022.102388 (PMC9530617; doi:10.1016/j.jbc.2022.102388)

**A**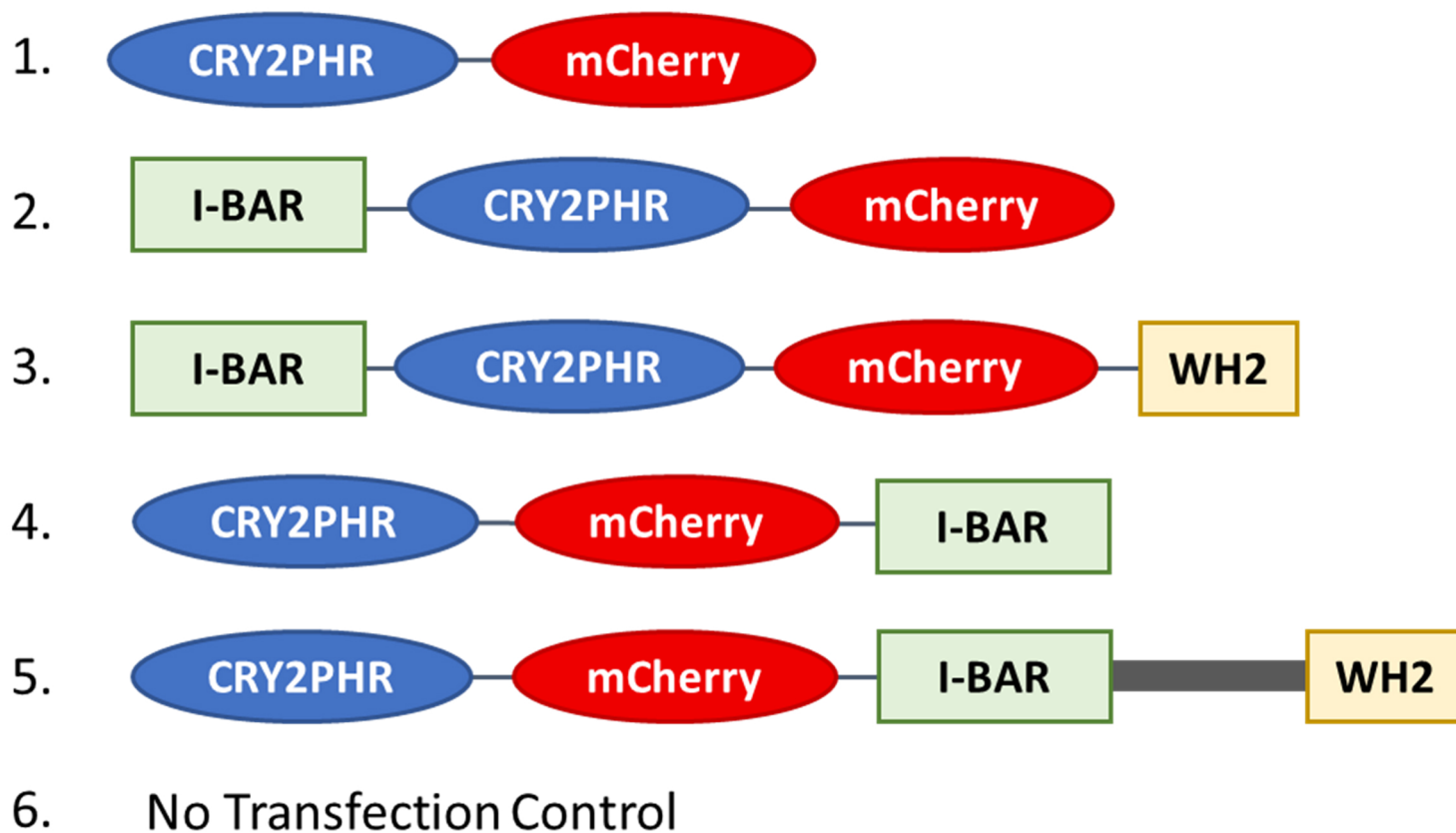**B**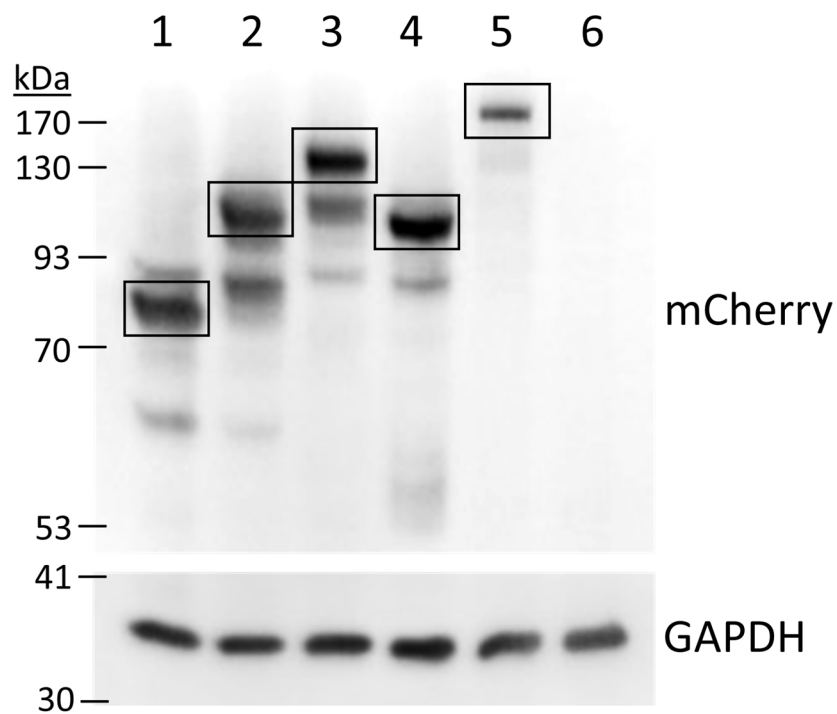

Supplement: Supplemental Figure S1 — CRY-BAR construct design and expression.A, diagrams of the CRY-BAR protein fusions; Constructs 2, 3, and 4 contain portions of the MTSS-1 protein (I-BAR, WH2, or both); Construct 5 contains the full length MTSS-1 protein. B, lysates of HEK293T cells transfected with CRY-BAR constructs and controls (Lanes 1–6, numbers correspond to panel A) were Western blotted with anti-mCherry antibody. Boxes indicate the apparent molecular weights: 1. 85 kDa; 2. 111 kDa; 3. 130 kDa; 4. 111 kDa; 5. 168 kDa; 6. No transfect control. The Western blot is representative of four independent trials [file mmc10.pdf]

**A**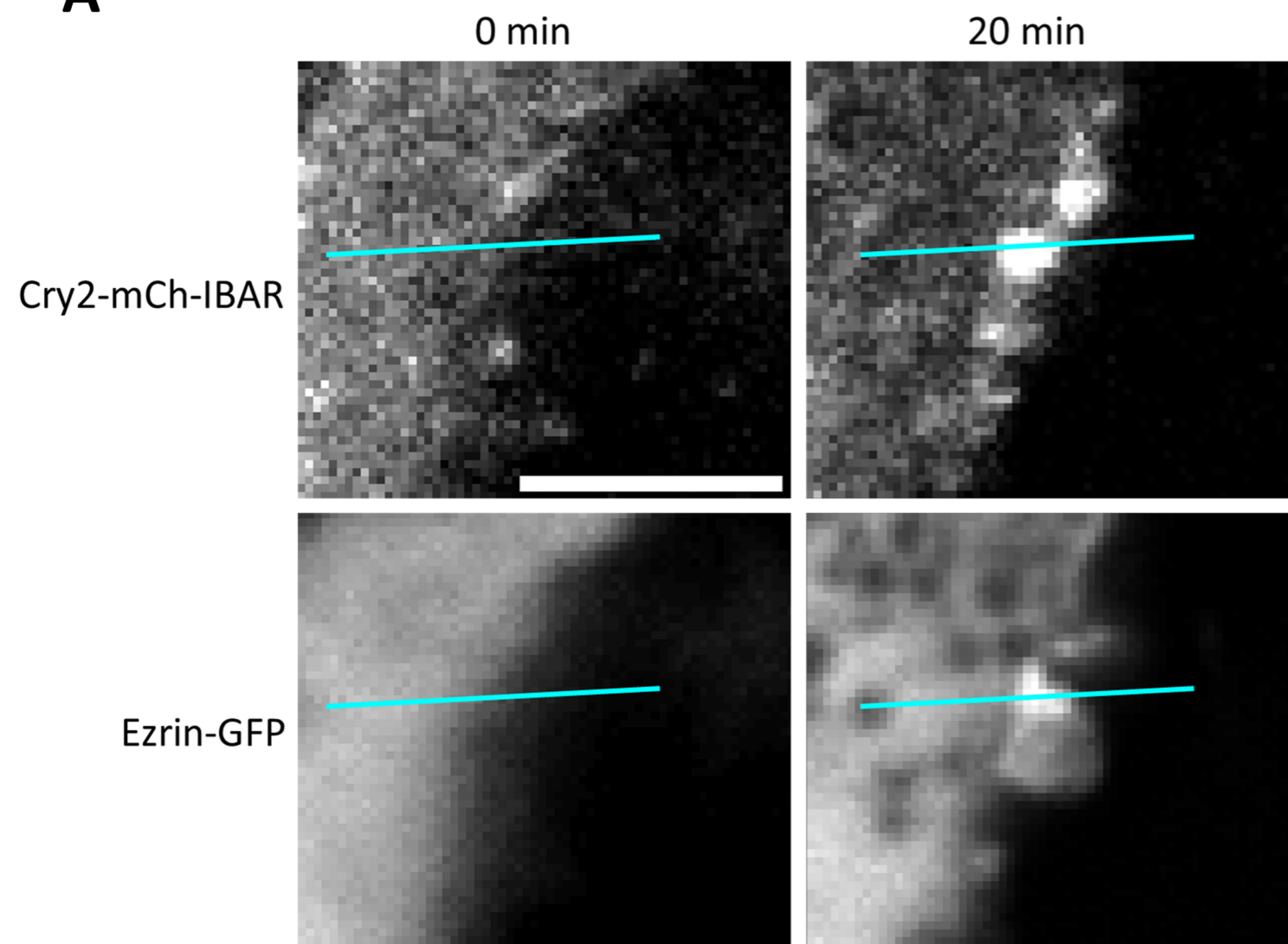**B**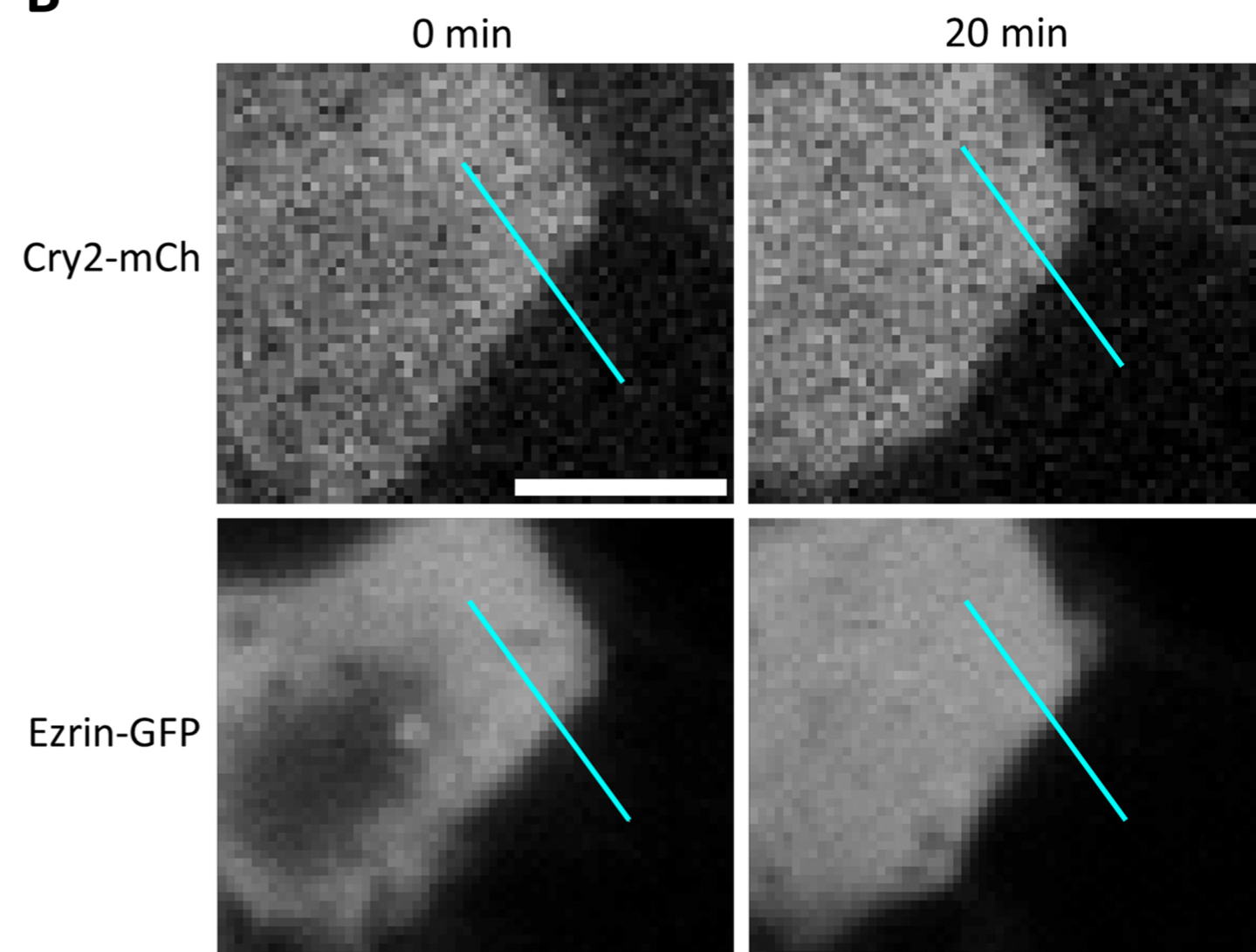**C**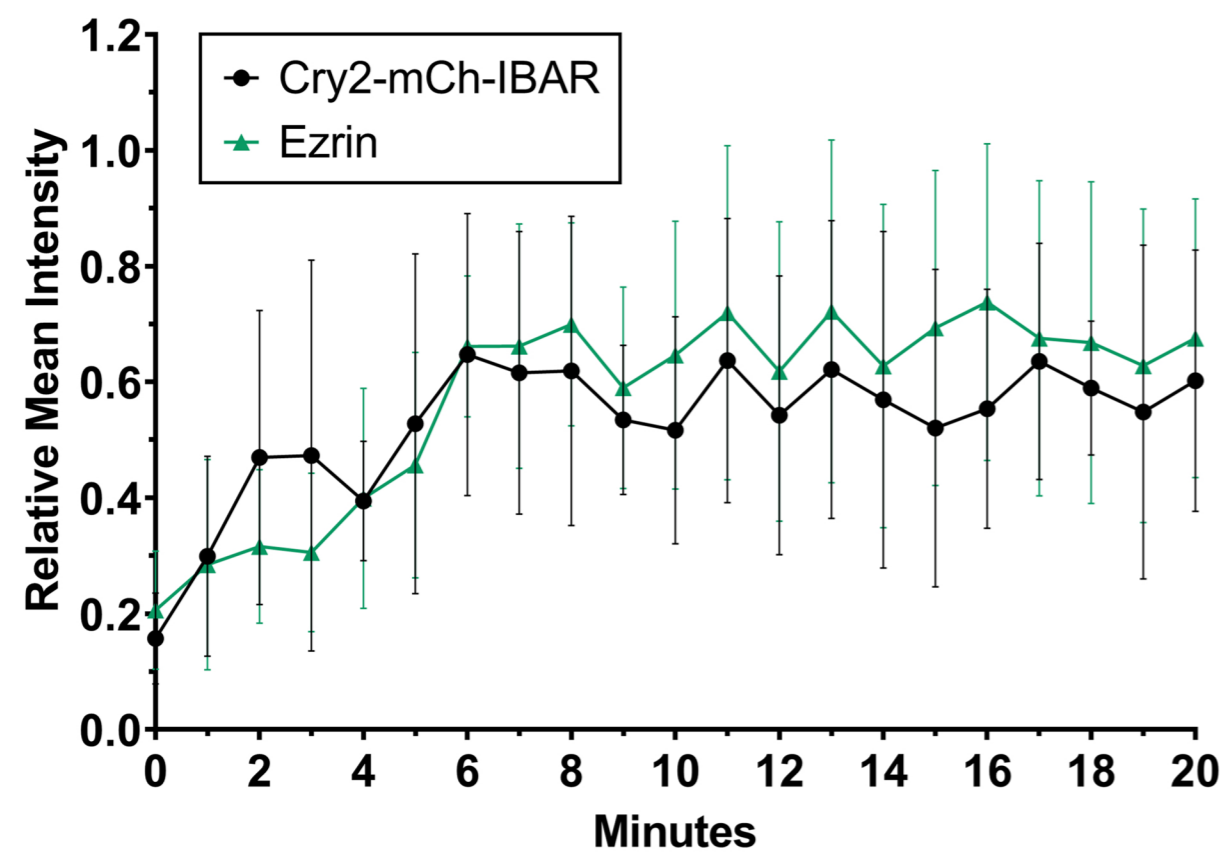**D**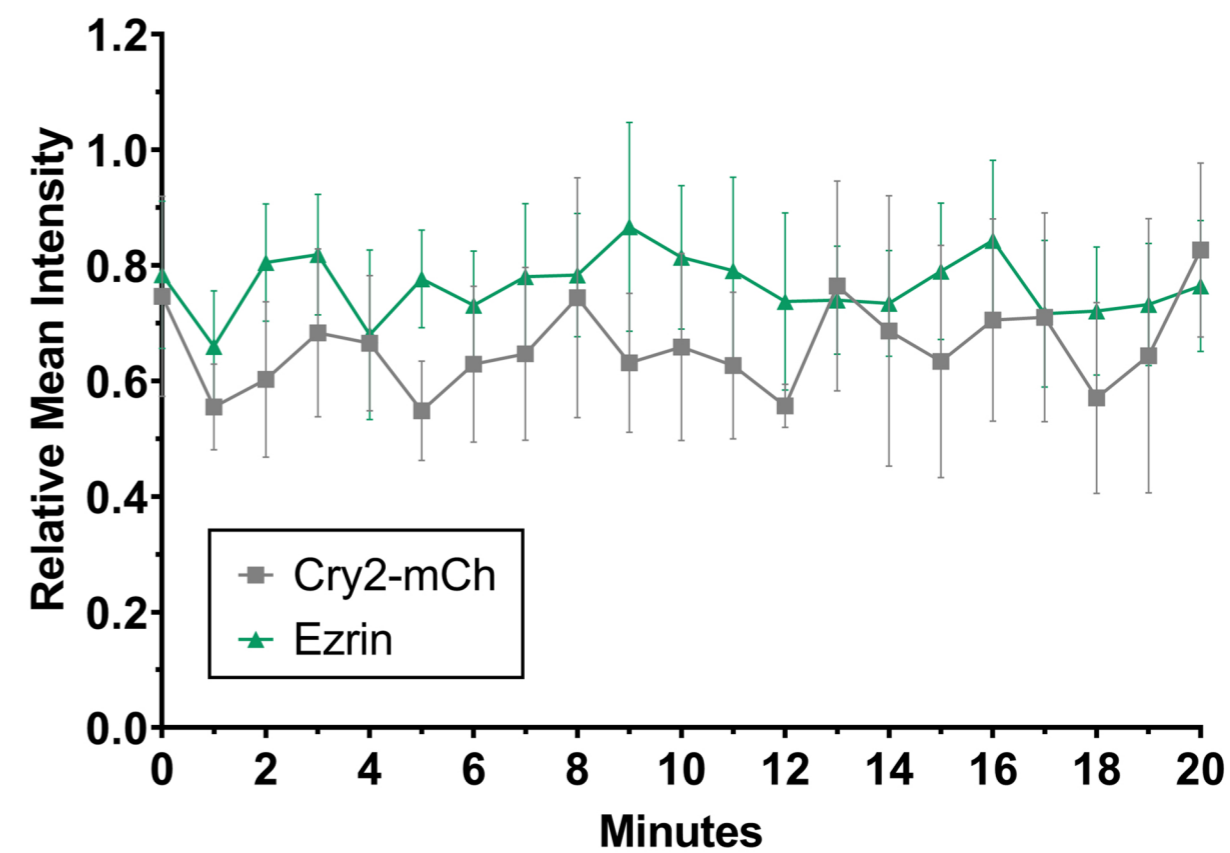

Supplement: Supplemental Figure S2 — Temporal relationship of Cry2-mCh-IBAR and Ezrin-GFP localization.A, HEK293T cells transfected with Cry2-mCh-IBAR and Ezrin-GFP were illuminated every 30 s with 488 nm light (5% power) on a confocal microscope over 20 min to assess their localization patterns in response to light activation of Cry2. Areas at the membrane exhibiting colocalization of Cry2-mCh-IBAR and Ezrin-GFP were analyzed for intensity over time by conducting a 6 micron line scan (cyan) ranging from the cytosol, through the membrane, to outside of the cell. Scale bar = 5 microns. B, HEK293T cells transfected with Cry2-mCh and Ezrin-GFP were imaged similarly to (A). Co-expressing cells were analyzed for intensity over time using a line scan (cyan) as described in (A). Line length: 6 microns. Scale bar = 5 microns. C, plot of intensity at the membrane (around 3 micron point of line) over the intensity in the cytosol (at the cytosolic end of line) from line analysis shown in (A). D, plot of intensity at the membrane (around 3 micron point of line) over the intensity in the cytosol (at the cytosolic end of line) from line analysis shown in (B). For (C) and (D), each point of intensity was taken at time 0 min of light exposure and then in 1 min increments for a total of 20 min (n = 6 cells). Membrane to cytosol ratios were normalized for intensity for each time point before averaging (error bars = standard deviation) [file mmc11.pdf]

**A**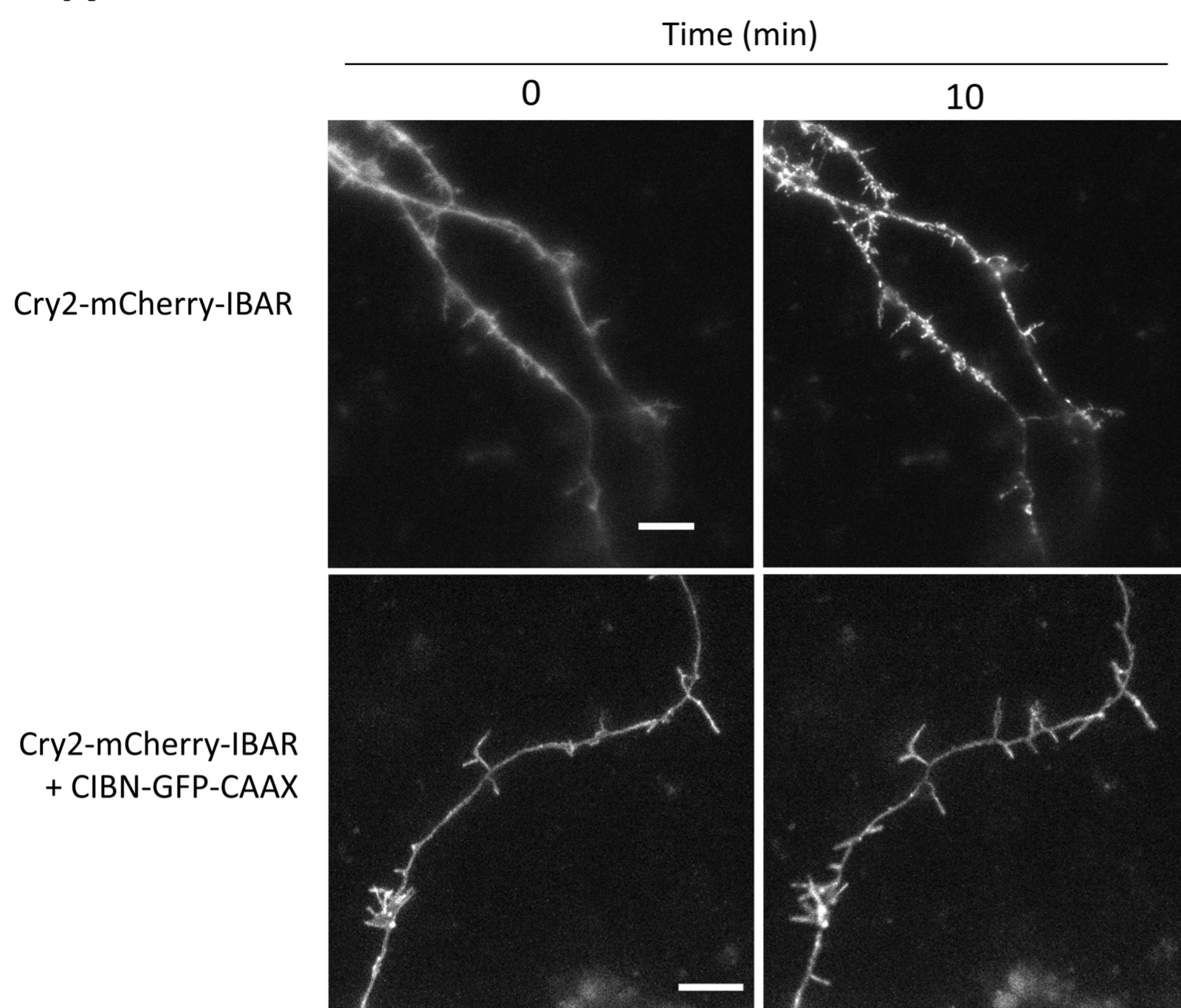**B**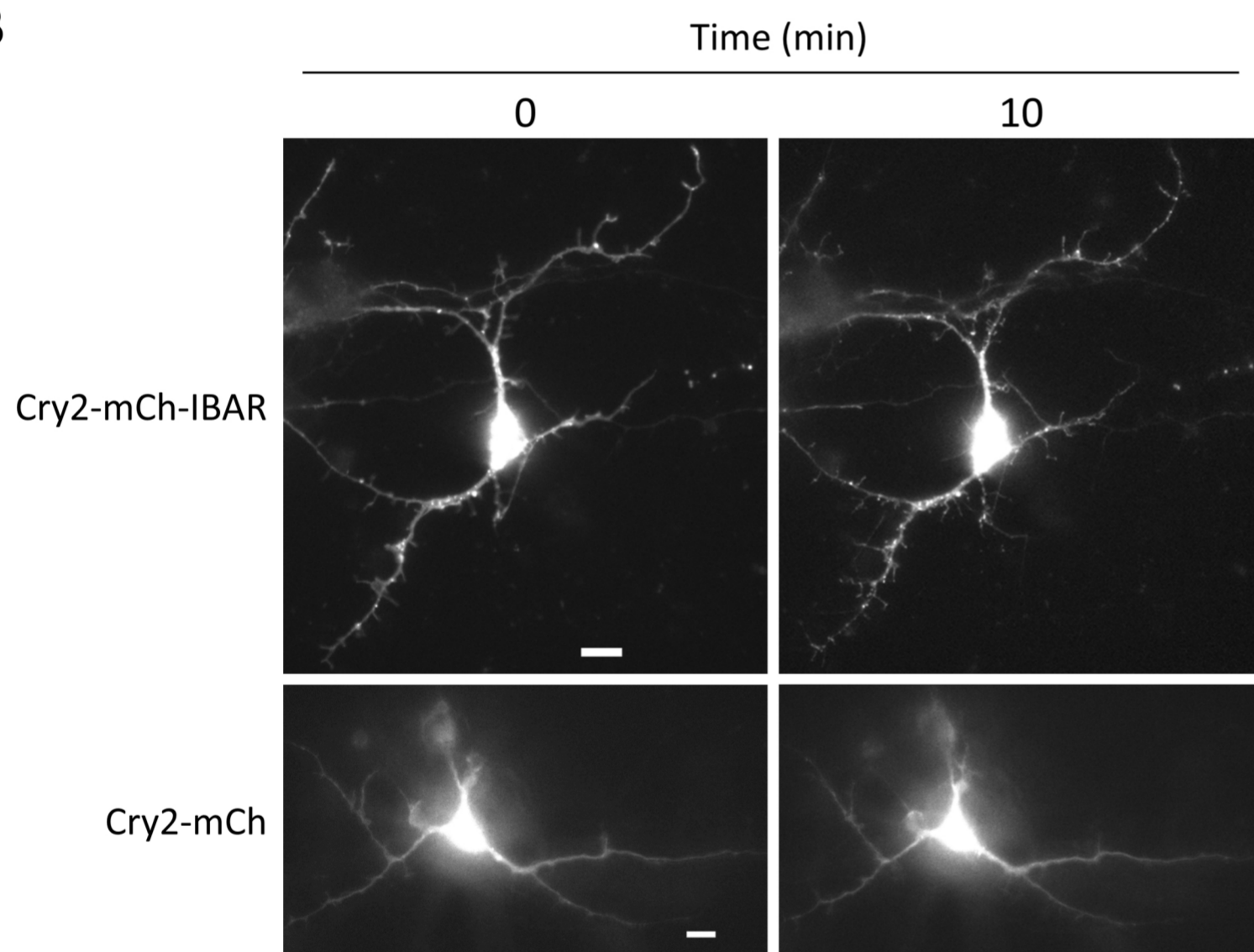**C**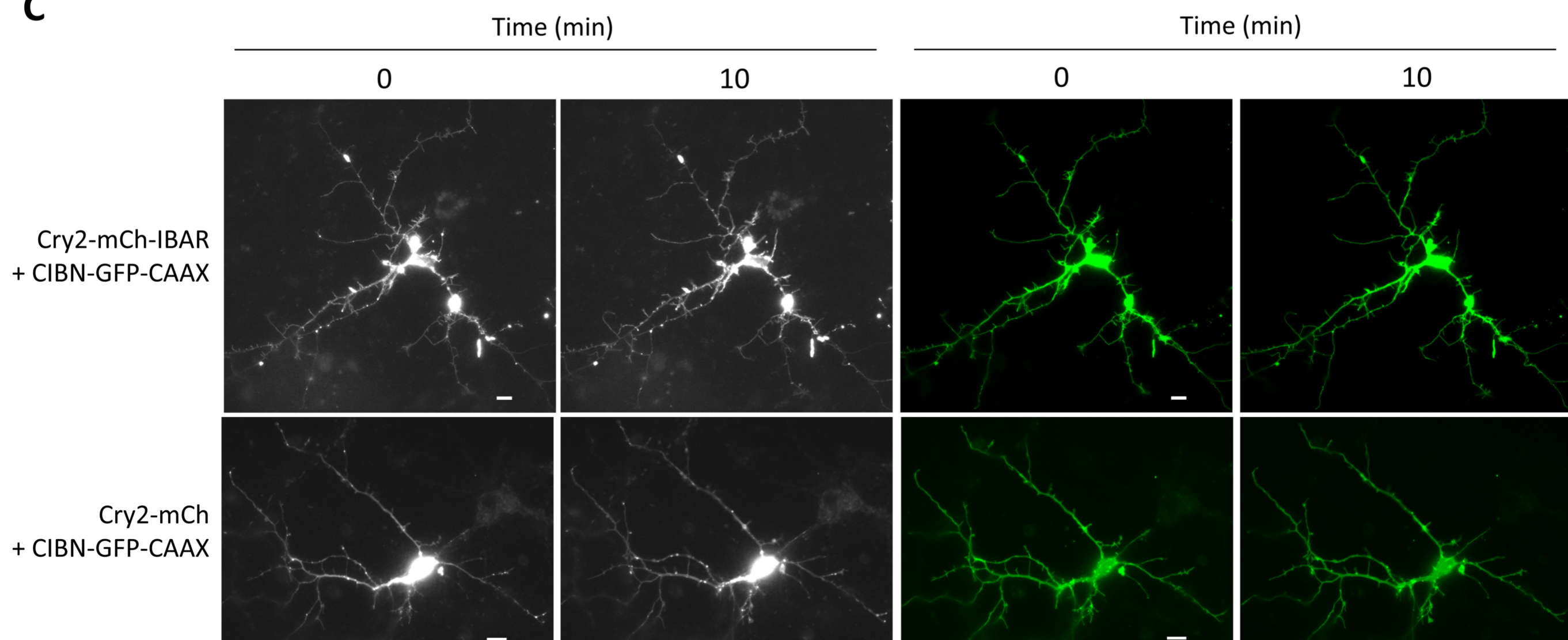

Supplement: Supplemental Figure S3 — Global activation of CRY-BAR in hippocampal neurons.A, neurons transfected with Cry2-mCh-IBAR; and co-transfected with Cry2-mCh-IBAR and CIBN-GFP-CAAX were illuminated every 30 s with 470 nm light on a widefield microscope. After 10 min, Cry2-mCh-IBAR forms numerous clusters throughout the neuronal processes, whereas in the presence of CIB, no such clusters are formed. Scale bar = 10 microns. B, full view of neurons transfected with Cry2-mCh-IBAR or Cry2-mCh. Neurons were illuminated every 30 s with 470 nm light on a widefield microscope. Scale bar = 10 microns. C, full view of neurons co-transfected with CIBN-GFP-CAAX (right; green) and Cry2-mCh-IBAR or Cry2-mCh (left; grey). Neurons were illuminated every 30 s with 470 nm light on a widefield microscope. Scale bar = 10 microns [file mmc12.pdf]

Cry2-mCherry-IBAR

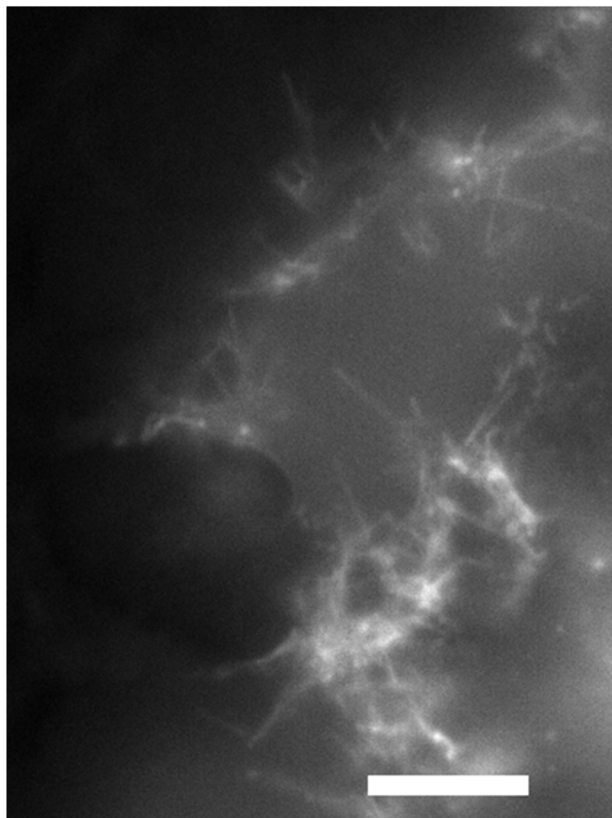

CIBN-GFP-CAAX

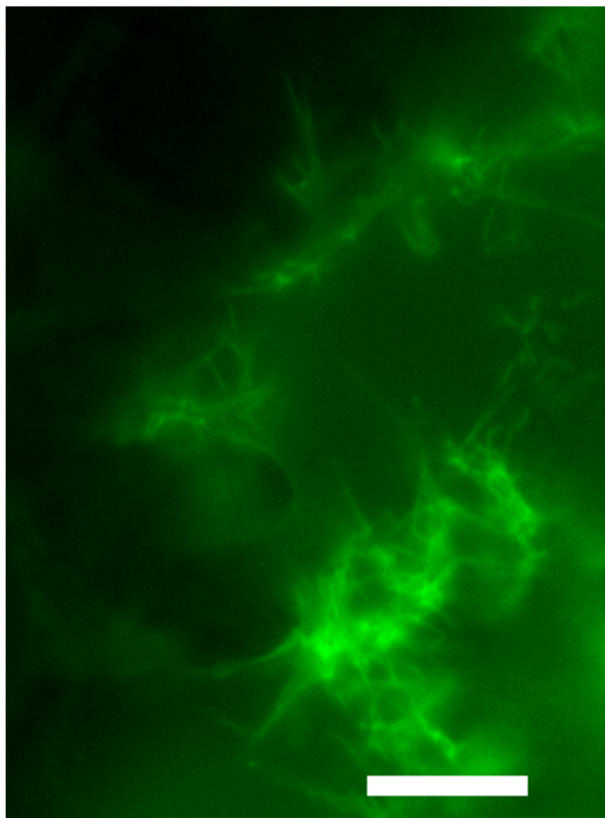

Overlay

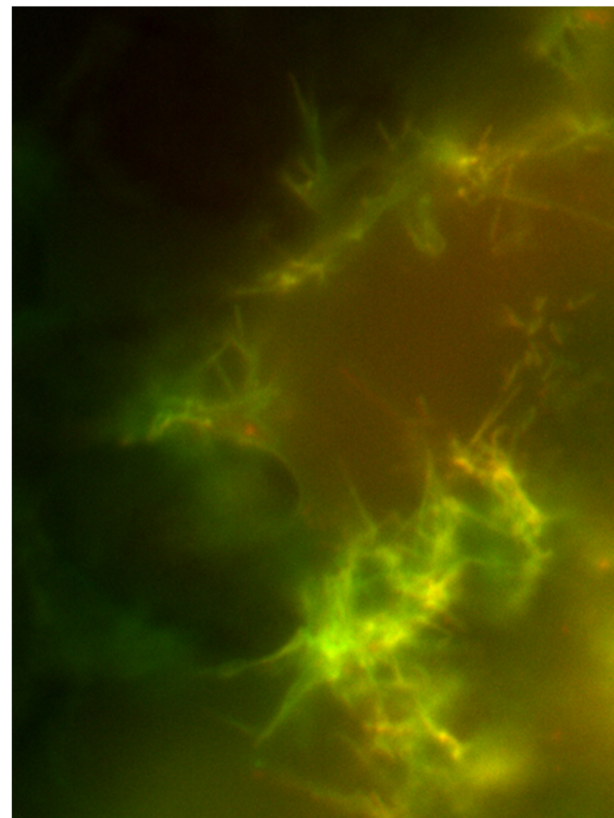

Supplement: Supplemental Figure S4 — Dark Localization of Cry2-mCh-IBAR and CIB-GFP-CAAX. HEK293T cells transfected with Cry2-mCh-IBAR and CIBN-GFP-CAAX were imaged sequentially (mCherry followed by GFP) to assess their localization patterns prior to light activation of Cry2. Both constructs have similar patterns of plasma membrane localization. Scale bar = 10 microns [file mmc13.pdf]
